# Dual Modulation of Lipid Metabolism by Adzuki Bean (*Vigna angularis*) Saponins Involving PPARα/γ Signaling and Gut Microbiota Remodeling in Obesogenic Diet-Induced Obese Mice

**DOI:** 10.3390/foods15183276

**Published:** 2026-09-16

**Authors:** Qingfeng Guo, Jinhai Luo, Xia Zhang, Hao Zhang, Meiru Chen, Man Liu, Zhenhua Yin, Juanjuan Zhang, Baocheng Yang, Li Wang, Baojun Xu, Lin Chen

**Affiliations:** 1 Henan Engineering Research Center of Chemistry and Biology of Medicinal Resources, Henan Comprehensive Utilization of Edible and Medicinal Plant Resources Engineering Technology Research Center, Zhengzhou Key Laboratory of Synthetic Biology of Natural Products, Henan Joint International Research Laboratory of Drug Discovery of Small Molecules, Huanghe Science and Technology College, Zhengzhou 450063, China; 2 Food Science and Technology Program, Department of Life Sciences, Beijing Normal-Hong Kong Baptist University, Zhuhai 519087, China; 3 School of Pharmacy, Henan University, Kaifeng 475004, China

**Keywords:** adzuki bean, obesity, triterpenoid saponin, PPARα/γ signaling, gut microbiota

## Abstract

Obesity is a prevalent metabolic disorder characterized by excessive lipid accumulation. This study aimed to investigate the anti-obesity effects and underlying mechanisms of two triterpenoid saponin-rich extracts, designated disaccharide saponin-rich extract (DTS) and trisaccharide/tetrasaccharide saponin-rich extract (TTS), prepared from adzuki bean (*Vigna angularis*). In vitro, both DTS and TTS extracts significantly inhibited lipid accumulation in 3T3-L1 adipocytes without cytotoxicity, with TTS reducing lipid droplets to 21.45% of the model group at 200 μg/mL. In vivo, C57BL/6J mice were fed a high-fat–high-cholesterol diet (HFD) for 12 weeks and orally administrated with DTS or TTS (30, 60, or 120 mg/kg/day) via gavage; the results showed marked reductions in body weight gain (up to 20.37%), fat mass, liver coefficient, and serum levels of total cholesterol (TC), triglyceride (TG), Low-Density Lipoprotein Cholesterol (LDL-C), Alanine Aminotransferase (ALT), and Aspartate Aminotransferase (AST), while elevating high-density lipoprotein cholesterol (HDL-C) levels. Mechanistically, transcriptomics and Western blot analyses revealed that both extracts were associated with upregulation of the PPARα-ACOX1 axis, consistent with enhanced fatty acid β-oxidation, and downregulation of the PPARγ-FABP4 pathway, consistent with reduced adipogenesis, suggesting a dual shift toward lipid catabolism and away from lipid synthesis. Furthermore, 16S rRNA sequencing demonstrated that both saponin extracts reversed HFD-induced gut dysbiosis by restoring α-diversity and orchestrating distinct genus-level modulations, specifically suppressing obesity-associated pathobionts (*Faecalibaculum* and *Ileibacterium*) while concurrently enriching beneficial commensals (*Bifidobacterium*). Notably, TTS at 120 mg/kg reduced body weight gain to a greater extent than the positive control orlistat (*p* < 0.05, Tukey’s HSD), with comparable improvements in serum lipid profiles and liver function parameters. Collectively, these findings demonstrate that adzuki bean saponin extracts exert anti-obesity effects in HFD-fed mice and are associated with multi-target changes involving the “liver–adipose–gut axis,” supporting their further evaluation as candidate as functional food ingredients for metabolic health.

## 1. Introduction

Obesity is a prevalent metabolic disorder characterized by excessive lipid accumulation, which has emerged as a major global public health challenge [1]. According to the Global Burden of Disease Study 2021, the global prevalence of overweight and obesity among adults has been rising steadily, with projections indicating continued increases through 2050. By 2050, the global population of overweight or obese adults is projected to reach 3.8 billion, representing over half of the expected adult population [2]. Obesity is closely associated with a cascade of metabolic complications, including insulin resistance, type 2 diabetes, non-alcoholic fatty liver disease, dyslipidemia, and cardiovascular diseases [3,4,5]. A high-fat–high-cholesterol diet (HFD) disrupts the balance of lipid metabolism, induces gut microbiota dysbiosis, and exacerbates metabolic disorders, making it one of the primary drivers of the obesity epidemic [6]. Therefore, improving dyslipidemia and reducing fat accumulation are key to preventing obesity-related metabolic diseases. Currently, treatment strategies for overweight and obesity include lifestyle changes, dietary modifications, pharmacological interventions, behavioral therapy, and surgery [7]. However, lifestyle and dietary interventions are often difficult to maintain long-term and are prone to rebound. Pharmacological agents such as orlistat and *L*-carnitine reduce lipid accumulation by promoting lipid breakdown, inhibiting intestinal absorption, and suppressing appetite, yet their long-term use is associated with significant side effects, including gastrointestinal discomfort and liver injury [8,9]. Consequently, the development of safe and effective intervention strategies based on natural food-derived bioactive components has attracted increasing attention.

The gut microbiota, a dynamic ecosystem, profoundly influences intestinal health and metabolic diseases [10]. Its metabolic activities, including carbohydrate fermentation and bile acid metabolism, regulate nutrient absorption and energy balance, linking it intimately to obesity. Dysbiosis contributes to obesity-related metabolic disorders and chronic inflammation, establishing gut microbiota as a potential target for obesity management [11]. Obesity has historically been associated with an altered Firmicutes/Bacteroidetes (F/B) ratio and a decline in beneficial bacteria such as *Bifidobacterium* [12], although the F/B ratio as a stand-alone marker of metabolic health is now considered controversial and is complemented in current analyses by genus- and function-level metrics [13]. Diet plays a crucial role in shaping gut microbiota; polyphenols and dietary fibers have been reported to modulate bacterial diversity and phylum-level community structure [14]. Microbiota-targeted strategies may improve insulin sensitivity, reduce hepatic steatosis, and promote adipose tissue browning [15].

Adipose tissue is a key regulator of whole-body energy homeostasis. Transcription factors such as peroxisome proliferator-activated receptor γ (PPARγ) and PPARα play central roles in controlling lipid storage and oxidation in a tissue- and context-dependent manner. PPARγ is predominantly expressed in adipocytes and regulates adipocyte differentiation, lipid accumulation, and insulin sensitivity; although its hepatic expression is low in the lean state, it is markedly upregulated during obesity, when it promotes hepatic de novo lipogenesis and steatosis [10]. Downstream of these transcription factors, key enzymes critically regulate lipid metabolic flux. Long-chain acyl-CoA synthetase 1 (ACSL1) catalyzes the activation of long-chain fatty acids to acyl-CoAs, directing them either toward esterification for storage or toward β-oxidation for energy production. Notably, ACSL1 expression is positively regulated by PPARγ, and its upregulation suppresses fatty acid oxidation while promoting triglyceride accumulation [16]. Acyl-CoA oxidase 1 (ACOX1), the rate-limiting enzyme of peroxisomal β-oxidation, is primarily responsible for the catabolism of very long-chain fatty acids (VLCFAs) and is transcriptionally activated by PPARα [17]. Thus, in the liver, the balance between PPARγ-driven storage-associated genes (e.g., ACSL1) and PPARα-driven oxidative genes (e.g., ACOX1) determines whether lipids are stored or oxidized, and dysregulation of this balance contributes to HFD-induced obesity and hepatic steatosis. Given that dietary saponins have been reported to modulate PPAR signaling and lipid metabolism [18], it is plausible that they exert anti-obesity effects by shifting this balance toward lipid oxidation via the PPARα/γ axis and its downstream enzymes.

In recent years, plant-derived saponins have garnered considerable attention for their anti-obesity properties. Saponins are a class of naturally occurring glycosides widely present in legumes, vegetables, and medicinal herbs. Numerous studies have demonstrated that dietary saponins exert anti-obesity effects through multiple mechanisms, including inhibition of pancreatic lipase activity to reduce intestinal fat absorption, modulation of adipocyte differentiation and lipid metabolism via the PPAR signaling pathway, activation of AMPK, and regulation of gut microbiota composition [19,20]. For example, ginsenosides from *Panax ginseng*, platycodin D from *Platycodon grandiflorum*, and escins from horse chestnut have all been reported to reduce body weight gain and improve lipid metabolism in HFD-fed animal models [21,22,23]. A comprehensive review on saponins in obesity management highlighted that saponin–lipase interaction and gut microbiota involvement in saponin metabolism are critical for their anti-obesity activity.

Adzuki bean (*V. angularis*), an annual leguminous plant native to East Asia, is rich in bioactive compounds including saponins, polyphenols, and polysaccharides [24]. It offers multiple health benefits, such as antioxidant, anti-inflammatory, and anti-obesity effects [25]. In particular, adzuki bean saponin extracts have been shown to reduce lipid accumulation in HepG2 cells and suppress adipogenesis in 3T3-L1 cells by activating the PI3K/Akt/GSK3β/β-catenin pathway and downregulating PPARγ and C/EBPα [26]. Black adzuki bean extract has likewise been shown to suppress PPARγ and FABP4 in differentiating 3T3-L1 adipocytes and to upregulate PPARα and ACOX expression in the adipose tissue of HFD-fed mice [27,28]. Recent studies have also demonstrated that dietary red bean seedlings extract alleviates obesity via activation of PPARα-AMPKα signaling in white adipose tissue [29]. Moreover, adzuki bean flavonoid mimics have been reported to ameliorate obesity and hepatic lipid metabolism abnormalities in HFD-induced obese mice by modulating sphingolipid metabolism and improving hepatic lipid composition [30]. Most recently, a combined transcriptomic and proteomic study of unfractionated adzuki bean saponins in HFD-fed mice identified soyasaponin Ba as a key active component [31]. Although these studies have demonstrated that adzuki bean saponin preparations can attenuate HFD-induced obesity in rodents, they have generally evaluated total saponin fractions or crude extracts as a single heterogeneous preparation. Whether saponin sub-fractions differing in sugar-chain length—such as the disaccharide saponin-rich extract (DTS) and the trisaccharide/tetrasaccharide saponin-rich extract (TTS)—differ in their regulation of lipid metabolism, energy homeostasis, and gut microbiota in vivo has not been directly compared within a single study.

In this study, we prepared two saponin-rich extracts from adzuki beans through a combination of ethanol extraction, solvent partitioning, and column chromatography. One extract, designated DTS, contained disaccharide saponins as its major tentatively annotated components, while the other extract, TTS, was enriched in tri- and tetrasaccharide saponins. To the best of our knowledge, these two saponin sub-fractions have not been directly compared under identical in vivo conditions. Although DTS and TTS are heterogeneous saponin fractions whose exact composition was not quantitatively defined, their distinct glycosylation profiles prompted us to compare their metabolic effects. Given that other plant-derived saponins show glycosylation-dependent activities, we speculated that DTS and TTS might exert differential regulation of lipid metabolism and the gut–liver axis. To test this hypothesis, we systematically evaluated the anti-obesity effects of DTS and TTS using both an HFD-induced obese mouse model and 3T3-L1 adipocytes. Specifically, we assessed body weight, serum lipid profiles, liver function, histopathological changes, and gut microbiota composition, and further investigated the regulatory effects of these saponin extracts on key genes and proteins involved in fatty acid oxidation and adipogenesis. This study provides a preclinical basis for evaluating adzuki bean-derived saponin extracts as candidate functional food ingredients, although further studies, including clinical trials, are required to determine their efficacy and safety in humans.

## 2. Materials and Methods

### 2.1. Materials and Reagent

Adzuki beans (*V. angularis)* originated from Hegang, Heilongjiang Province, China, and were obtained from Luxianda Biotechnology Co., Ltd. (Jining, China). Trypsin solution (0.25%) and penicillin–streptomycin solution were purchased from HyClone; 3-isobutyl-1-methylxanthine (IBMX) and dexamethasone (DEX) were procured from Sigma-Aldrich Co., Ltd. (St. Louis, MO, USA). Dulbecco’s Modified Eagle Medium (DMEM) and insulin were supplied by Beijing Maichen Technology Co., Ltd., while dimethyl sulfoxide (DMSO) and the Oil Red O Staining Kit were obtained from Solarbio (Beijing, China). For cell-based experiments, anti-PPARα, anti-PPARγ, anti-ACOX1, anti-ACSL1, anti-FABP4, and anti-β-actin antibodies were purchased from Proteintech (Wuhan, China); for animal experiments, the same set of antibodies was provided by Servicebio (Wuhan, China). The BCA Protein Assay Kit was sourced from PU XI-BIO (Shanghai, China).

### 2.2. Preparation of DTS, TTS Extracts

The powdered seeds of *V. angularis* (30.0 kg) were extracted with 70% EtOH (70 L) at room temperature for 48 h, repeated three times. The combined extracts were concentrated to yield ~1.0 kg of residue, which was suspended in H_2_O (5 L) and partitioned successively with petroleum ether, EtOAc, and *n*-BuOH (each 3×, equal volume). The *n*-BuOH fraction (206.0 g) was loaded onto AB-8 resin and eluted stepwise with 0%, 20%, 50%, 70%, and 95% EtOH to give fractions A–E. Fraction D (72 g) was chromatographed on silica gel (100–200 mesh) with CH_2_Cl_2_–MeOH–H_2_O (9:2:0.1 → 8:2:0.2 → 7:3:0.5) to afford subfractions D1–D5. D2 was purified by recrystallization from MeOH (3–5 cycles) to give a triterpenoid saponin mixture (a trisaccharide/tetrasaccharide saponin-rich extract, designated as TTS-A, 4.5 g) as a pale yellow powder. D5 was further eluted with CH_2_Cl_2_–MeOH–H_2_O (9:2:0.2 → 8:2:0.3 → 7:3:0.5), yielding D-5-2 (a disaccharide triterpenoid saponin-rich extract, DTS, 7.3 g) and D-5-3 (a trisaccharide/tetrasaccharide saponin-rich extract, designated as TTS-B, 2.9 g). All fractions were monitored using TLC and LC–MS. Since TTS-A and TTS-B showed similar LC–MS profiles and were individually insufficient for the full in vivo study, they were pooled at a 4.5:2.9 mass ratio and used as the TTS fraction in all subsequent experiments.

### 2.3. Analysis of DTS and TTS by HPLC-DAD-ESI-MS

The chemical profiles of the triterpenoid saponin extracts (DTS and TTS) from *V. angularis* were analyzed using high-performance liquid chromatography coupled with a diode-array detector and electrospray ionization mass spectrometry using an Agilent 6400 Series Triple Quadrupole LC/MS System (Agilent Technologies, Inc., Santa Clara, CA, USA) (HPLC-DAD-ESI-MS). Chromatographic separation was performed on an Agilent Poroshell Plus C18 column (3.0 mm × 50 mm, 2.7 µm) at 40 °C. The mobile phase consisted of water containing 0.05% formic acid (FA) (A) and acetonitrile containing 0.05% FA (B), with a gradient elution program: 0–5 min, 5–25% B; 5–20 min, 25–75% B; 20–24 min, 75–95% B. The flow rate was 0.3 mL/min, and the injection volume was 5 µL. UV detection was set at 205 nm with a reference wavelength of 360 nm. Mass spectra were acquired in negative-ion mode with an ESI source. The fragmentor voltage was 120 V, desolvation gas (N_2_) flow rate was 12 L/min, and ionization temperature was 345 °C. Full-scan mass spectra were recorded over the *m/z* range of 100–1500. All parameters were controlled by the Agilent 6400 Series system software.

### 2.4. In Vitro Experiments

#### 2.4.1. Cell Culture and Adipocyte Differentiation

3T3-L1 preadipocytes, sourced from Procell Life Science & Technology Co., Ltd. (Wuhan, China), were routinely maintained in DMEM enriched with 10% heat-inactivated fetal bovine serum (FBS) and 1% penicillin/streptomycin, and incubated at 37 °C in a humidified atmosphere with 5% CO_2_. To initiate adipogenic differentiation, cells cultured to two days post-confluence (designated as Day 0) were treated with differentiation medium I (MDI: DMEM containing 0.5 mmol/L IBMX, 1 μmol/L dexamethasone, and 10 μg/mL insulin). On Day 2, the medium was exchanged for differentiation medium II (DMEM supplemented with 10 μg/mL insulin), which was then refreshed every 48 h starting from Day 4. By the end of the 8-day induction period, over 80% of the cell population had acquired a mature adipocyte phenotype, as confirmed by prominent intracellular lipid droplet formation [32].

#### 2.4.2. MTT Assay for Cell Viability

With reference to a previously established protocol [33] with minor modifications, 3T3-L1 preadipocytes in the logarithmic growth phase were seeded into 96-well plates at a density of 5 × 10^3^ cells/well and allowed to attach overnight. The cells were subsequently incubated for 48 h with varying concentrations (10, 50, 100, and 200 μg/mL) of the test compound. Following treatment, the culture medium was removed, the wells were washed three times with PBS, and 100 μL of fresh medium containing 0.5 mg/mL MTT was added to each well for a 4-h incubation. The supernatant was then carefully aspirated, and the resulting formazan crystals were dissolved in 100 μL of DMSO with agitation for 10 min. Finally, the optical density was recorded at 570 nm using a microplate reader.

#### 2.4.3. Drug Treatment and Oil Red O Staining

From the onset of differentiation induction (day 0), cells were treated with graded concentrations of the two test samples—DTS (50 and 100 μg/mL) and TTS (100 and 200 μg/mL)—supplemented into the differentiation medium. The chosen doses were validated by MTT assays, which confirmed >95% cell viability. Untreated preadipocytes and differentiated adipocytes served as negative and positive controls, respectively. On day 8, the medium was removed, and cells were rinsed twice with PBS, fixed in 4% Oil Red O fixative for 20–30 min at ambient temperature, and quickly washed with 60% isopropanol. Staining was performed by adding freshly prepared Oil Red O working solution for 10–20 min, after which cells were washed with 60% isopropanol until clear intercellular spaces appeared, followed by 2–5 distilled-water rinses to eliminate excess dye. Nuclear counterstaining was carried out with Mayer’s hematoxylin for 1–2 min; after washing, slides were incubated in Oil Red O buffer for 1 min to achieve blueing, then mounted under distilled water for microscopy. For quantitative assessment, the stained dye was eluted with 100% isopropanol under gentle shaking for 10 min, and absorbance was recorded at 510 nm using a microplate reader.

#### 2.4.4. RNA Extraction and Rt-qPCR

Total RNA was isolated from differentiated 3T3-L1 adipocytes using TRNzol Universal reagent (DP424, Tiangen, Beijing, China) following the kit protocol. Cells were lysed directly in culture dishes, and the homogenates were transferred to RNase-free tubes. RNA purity and concentration were assessed with a Nanodrop 2000 spectrophotometer (Thermo Scientific, Wilmington, DE, USA). For cDNA synthesis, equal amounts of total RNA were reverse-transcribed using the RevertAid First Strand cDNA Synthesis Kit (K1622, Thermo Scientific, Wilmington, DE, USA) on a Veriti PCR instrument (ABI, Foster City, CA, USA). Quantitative real-time PCR (qRT-PCR) was performed on an ABI 7500 system (ABI, Foster City, CA, USA) with 2× SYBR Green qPCR Master Mix (B21203, Selleck, Houston, TX, USA). The thermal cycling protocol comprised an initial denaturation step followed by repeated cycles of denaturation and annealing/extension; melting curve analysis was included to confirm amplicon specificity. Relative transcript levels were calculated using the 2^−ΔΔCt^ method, with GAPDH as the endogenous reference. All primer sequences are provided in Supplementary Table S1.

#### 2.4.5. Western Blot Analysis for Cell Samples

Total protein was extracted using RIPA lysis buffer (Jinke Biotechnology, Chongqing, China) containing PMSF (Merck Millipore, Burlington, MA, USA). After washing with ice-cold PBS, cells were lysed on ice for 30 min and centrifuged at 12,000 rpm for 10 min at 4 °C, and the supernatant was collected. Protein concentrations were determined with a BCA kit (Jinke, Chongqing, China) on a SpectraMax M5 (Molecular Devices, San Jose, CA, USA). Equal amounts (80 μg) of denatured proteins (boiled with loading buffer) were resolved by SDS-PAGE at 140 V, transferred to PVDF membranes (semi-dry, 200 mA, 1 h), blocked with 5% non-fat milk in TBST (1 h), and probed with primary antibody (1:1000, overnight at 4 °C) followed by HRP-conjugated secondary antibody (1:3000, 1 h). Bands were visualized with ECL Plus (Biosharp, Beijing, China) and imaged on a Tanon 5200 system (Shanghai Tanon Science & Technology Co., Ltd., Shanghai, China). Intensities were quantified using Gelpro32 and normalized to β-actin. Antibody details are listed in Supplementary Table S2.

### 2.5. In Vivo Experiments

#### 2.5.1. Animal Model and Experimental Design

Male C57BL/6J mice (7 weeks old, *n* = 72) were obtained from Henan Zhongge Biotechnology Co., Ltd. (Kaifeng, China). All animal procedures were approved by the Animal Ethics Committee of Huanghe University of Science and Technology (Approval No. 2025-046, 2 July 2025) and were conducted in accordance with the National Institutes of Health Guide for the Care and Use of Laboratory Animals. Upon arrival, mice were acclimatized for one week under controlled environmental conditions (24 ± 2 °C, 50–70% relative humidity, 12-h light/dark cycle) with free access to standard chow and water. After acclimatization, animals were randomly divided into nine groups (*n* = 8 per group) using SPSS 21.0 software, with mice from the same cage distributed across different treatment arms to control for cage-cluster effects. This group size is consistent with established practice in C57BL/6J diet-induced obesity studies [28,31] and complies with the ARRIVE 2.0 guidelines and the 3R principle. The groups were divided as follows: the normal control (NC) group received a standard diet and 0.5% carboxymethyl cellulose sodium (CMC-Na) solution (vehicle); the Model group received an obesogenic high-fat–high-cholesterol experimental diet (HFD) and vehicle; the DTS-L, DTS-M, and DTS-H groups received HFD supplemented with DTS at 30, 60, or 120 mg/kg/day, respectively; the TTS-L, TTS-M, and TTS-H groups received HFD supplemented with TTS at 30, 60, or 120 mg/kg/day, respectively; and the OT group received HFD and orlistat (30 mg/kg/day, suspended in 0.5% CMC-Na). All treatments were administered by daily oral gavage (10 mL/kg body weight) for 12 consecutive weeks, with vehicle given to the Control and Model groups. Body weight was recorded weekly for each individual mouse; food intake was recorded weekly per cage and averaged per mouse to obtain cage-level means for statistical analysis. At the end of the experimental period, mice were fasted for 12 h with free access to water, weighed, and anaesthetized. Blood was collected via retro-orbital puncture, allowed to clot at room temperature for 1–2 h, centrifuged at 4 °C and 4500 rpm for 10 min to obtain serum, and stored at −80 °C for subsequent analyses. Tissues—including white adipose (perirenal and epididymal), brown adipose (interscapular), and liver—were rapidly excised, rinsed with saline, weighed, and divided into two portions: one fixed in 4% paraformaldehyde for histological examination, and the other snap-frozen in liquid nitrogen and stored at –80 °C for further analyses. Diet compositions and energy contents are provided in Supplementary Table S3.

#### 2.5.2. Oral Glucose Tolerance Test

Following an overnight fast (12 h), fasting blood glucose levels were determined. Glucose was then administered orally at a dose of 2 g/kg body weight. Blood glucose concentrations were measured at 0, 15, 30, 60, and 120 min post-administration using a commercial glucometer (Sinocare Inc., Changsha, China). The area under the glucose curve (AUC) was calculated using GraphPad Prism software 10.1 (GraphPad Software, San Diego, CA, USA).

#### 2.5.3. Biochemical Analysis

Plasma levels of triglycerides (TG), total cholesterol (TC), high-density lipoprotein cholesterol (HDL-C), low-density lipoprotein cholesterol (LDL-C), aspartate aminotransferase (AST), and alanine aminotransferase (ALT) were measured using assay kits from the Nanjing Jiancheng Bioengineering Institute (Nanjing, China), following the manufacturer’s instructions.

#### 2.5.4. Histopathological Analysis

Epididymal white adipose tissue (WAT), brown adipose tissue (BAT), and liver samples were fixed in 10% neutral buffered formalin, embedded in paraffin, and sectioned at 4 μm thickness. For histological evaluation of general morphology and adipocyte size, sections were stained with haematoxylin and eosin (H&E) using a commercial kit (Servicebio, G1076, Wuhan, China). For lipid visualization, frozen sections were stained with Oil Red O (Servicebio, G1015, Wuhan, China) according to the manufacturer’s instructions. Briefly, sections were fixed in 10% formalin, rinsed with 60% isopropanol, and incubated with freshly prepared Oil Red O working solution for 30 min. After washing with distilled water, nuclei were counterstained with haematoxylin. All stained sections were examined and photographed under a light microscope.

#### 2.5.5. Quantitative Real-Time Polymerase Chain Reaction (qRT-PCR) Analysis of Liver mRNA Expression

Total RNA was isolated from mouse liver tissue using the RNA extraction reagent (G3013, Servicebio, China). Frozen samples were homogenized in the reagent with zirconia beads on a tissue grinder (KZ-5F-3D, Servicebio). After centrifugation, the supernatant was collected and subjected to chloroform substitution, vortexing, and further centrifugation for phase separation. The aqueous phase was precipitated with isopropanol at low temperature; the RNA pellet was washed with ethanol, air-dried, and dissolved in RNA dissolution buffer. RNA concentration and purity were assessed with a Nanodrop 2000 spectrophotometer (Thermo Scientific). For cDNA synthesis, total RNA was reverse-transcribed using the SweScript All-in-One RT SuperMix kit (G3337, Servicebio), with a reaction mixture containing 5× SuperMix, gDNA Remover, RNA template, and nuclease-free water, run on a Veriti PCR instrument (ABI) under sequential temperature conditions. Quantitative real-time PCR was performed on a CFX Connect system (Bio-Rad, Hercules, CA, USA) using 2× Universal Blue SYBR Green Master Mix (G3326, Servicebio). Each reaction included Master Mix, primers, cDNA, and water. The thermal protocol comprised initial denaturation followed by cycles of denaturation and annealing/extension, with melting curve analysis to verify specificity. Relative gene expression was calculated using the 2^−ΔΔCt^ method with GAPDH as the internal control. Primer sequences are given in Supplementary Table S4.

#### 2.5.6. Protein Extraction and Western Blotting

Total protein was extracted from liver tissue and quantified using a BCA protein assay kit (Servicebio, G2026). Equal amounts of protein were resolved by SDS-PAGE on appropriate gel concentrations and transferred onto PVDF membranes (Servicebio, G6047). After blocking, membranes were incubated overnight at 4 °C with primary antibodies against the target proteins (see Supplementary Table S5 for details), followed by incubation with the corresponding HRP-conjugated secondary antibody (also listed in Table S2). Protein bands were visualized using an enhanced chemiluminescence (ECL) kit (Servicebio, G2020 or G2074) and a chemiluminescence imaging system (Servicebio, SCG-W3000 PLUS). Band intensities were quantified with AIWBwell™ analysis software (version 1.0; Servicebio, Wuhan, China) and normalised to β-actin, which was confirmed to be stably expressed across all samples and used as the loading control. All experimental procedures, including animal handling, sample collection, and molecular analyses, were performed following the protocols previously described [34].

#### 2.5.7. RNA Sequencing and Transcriptome Analysis

Total RNA was extracted from tissue samples from *n* = 4 biologically independent mice per group (9 groups sequenced; total = 36 libraries) using TRIzol reagent (Servicebio, Wuhan, China). Paired-end sequencing libraries were constructed with the mRNA-seq library preparation kit (Servicebio) following the manufacturer’s protocol, and library quality was assessed using an Agilent TapeStation 4150 system (Agilent Technologies, Santa Clara, CA, USA). The libraries were sequenced on an Illumina NovaSeq 6000 platform (Servicebio, China). Raw FASTQ reads were preprocessed with fastp for quality control, including adapter trimming, quality filtering, and per-read base trimming, to obtain clean reads. The clean reads were aligned to the reference genome using STAR v2.7.8a. Gene expression levels were quantified with StringTie v2.2.1, and transcript abundance was calculated as transcripts per million (TPM). Differential expression analysis between groups was performed using the DESeq2 R package (v1.42.1); genes with |log_2_(Fold Change)| > 1 and an adjusted *p*-value < 0.05 (Benjamini–Hochberg correction) were considered significantly differentially expressed. Functional enrichment analyses, including Gene Ontology (GO) and KEGG pathway annotations, were carried out using the clusterProfiler R package (v4.10.1) [35]. All sequencing services were provided by Servicebio (Wuhan, China).

### 2.6. 16S rRNA Sequencing and Gut Microbiota Analysis

Faecal microbial genomic DNA was extracted using the MGIEasy Stool Genomic DNA (meta) Extraction Kit (MGI Tech, Shenzhen, China) following the manufacturer’s instructions. The V3–V4 hypervariable region of the bacterial 16S rRNA gene was amplified by PCR using the fusion primers 338F (5′-ACTCCTACGGGAGGCAGCAG-3′) and 806R (5′-GGACTACHVGGGTWTCTAAT-3′); PCR products were purified with AMPure XP beads, and libraries were quality-checked on an Agilent 2100 Bioanalyzer (Agilent Technologies, Santa Clara, CA, USA). Paired-end sequencing (2 × 300 bp, target 50,000 tags/sample) was performed on the BGI Genomics platform (Shenzhen, China). A total of 45 faecal samples (9 groups, *n* = 5 per group) were sequenced. Raw reads were trimmed with cutadapt v2.6, and low-quality reads were filtered using a sliding window (30 bp, Q20); reads <75% of original length, containing ambiguous bases, or with low complexity were discarded. Clean reads were assembled into Tags using FLASH v1.2.11 (minimum overlap 15 bp, mismatch 0.1). OTUs were clustered at 97% similarity with USEARCH v11.0.667 (UPARSE), and chimeras removed with UCHIME v4.2.40 against the gold reference database (v20110519). Taxonomy was assigned using the RDP classifier v1.9.1 (confidence ≥ 0.6) against the RDP database (Release 19, 20230720). Samples were rarefied to the minimum number of tags per sample to normalize sequencing depth. Alpha diversity was assessed using the Observed species, ACE, Chao1, Shannon, and Simpson indices. Beta diversity was evaluated by partial least squares discriminant analysis (PLS-DA) based on Bray–Curtis dissimilarity, and differentially abundant taxa were identified by linear discriminant analysis effect size (LEfSe) analysis (LDA > 2, *p* < 0.05). All bioinformatic analyses were performed using the BGI online microbiome analysis platform (https://meta.bgi.com (accessed on 7 July 2026)).

### 2.7. Statistical Analysis

Statistical analyses were performed using GraphPad Prism 10.1 (GraphPad Software, San Diego, CA, USA). Data are expressed as mean ± standard error of the mean (SEM). Body weight (recorded weekly per mouse) and food intake (recorded per cage and averaged per mouse), both measured weekly throughout the 12-week intervention, were analyzed using linear mixed-effects models (LMMs) with treatment, time (week), and treatment × time as fixed effects, and mouse (or cage, for food intake) as a random effect; sphericity was assessed using Mauchly’s test with Greenhouse–Geisser correction applied when violated, and Bonferroni-corrected pairwise comparisons were performed when the treatment × time interaction was significant. Comparisons among multiple groups were assessed by one-way ANOVA followed by Dunnett’s post hoc test, as appropriate. Direct comparisons between DTS and TTS at matched dose levels were performed within the same one-way ANOVA model using Tukey’s HSD post hoc test. Differences between two groups were evaluated using an unpaired two-tailed Student’s *t*-test. A *p*-value < 0.05 was considered statistically significant. Significance levels are indicated as * *p* < 0.05, ** *p* < 0.01, and *** *p* < 0.001 versus the control group or as specified in the figure legends.

## 3. Results

### 3.1. LC-MS Profiling and Identification of Bioactive Saponins in DTS and TTS Extracts from Adzuki Beans

**Figure 1:**
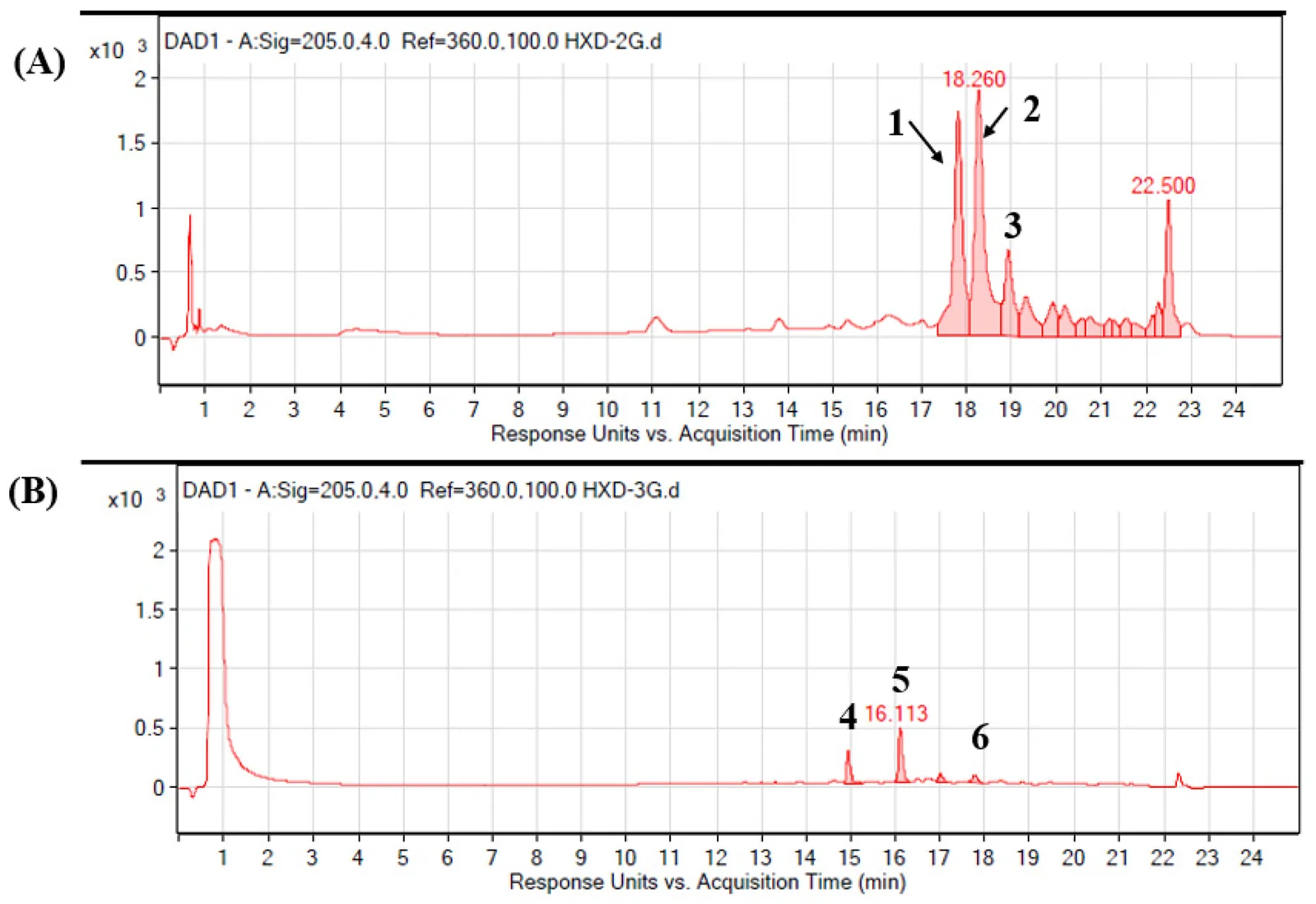
The representative base peak ion chromatograms chromatogram of DTS (**A**) and TTS (**B**) extract in negative ion mode. Peaks were annotated as follows: 1. Angulasaponin A, 2. azukisaponin II, 3. azukisaponin I, 4. azukisaponin IV, 5. azukisaponin VI, 6. yunganoside B1.

Under the established HPLC-DAD-ESI-MS conditions, the DTS and TTS extracts were analyzed, and their chromatographic profiles at 205 nm are shown in Figure 1A,B, respectively. Because authentic reference standards were not available, all compound assignments are tentative and based on retention times, deprotonated molecular ions, and characteristic MS/MS fragmentations, with reference to literature data [36,37] and the mass spectra presented in Supplementary Figure S1.

In the total ion chromatogram (TIC) of the DTS extract, three major peaks were detected at retention times of 18.12, 18.26, and 19.39 min, giving deprotonated ions at *m*/*z* 793.1, 795.1, and 779.4 [M − H]^−^, respectively. These peaks were tentatively assigned as angulasaponin A, azukisaponin II, and azukisaponin I [36,37].

For the TTS extract, three major TIC peaks were observed at retention times of 15.17, 16.11, and 17.90 min, with deprotonated ions at *m*/*z* 971.3, 1133.4, and 941.3 [M − H]^−^, respectively. On the basis of the MS data and literature comparison [36,37], these were assigned to azukisaponin IV, azukisaponin VI, and yunganoside B1. According to the reported structures, azukisaponin IV and yunganoside B1 are reported to be trisaccharide saponins, whereas azukisaponin VI is reported to be a tetrasaccharide saponin.

### 3.2. DTS and TTS Inhibit Adipogenesis and Lipid Accumulation in 3T3-L1 Cells

**Figure 2:**
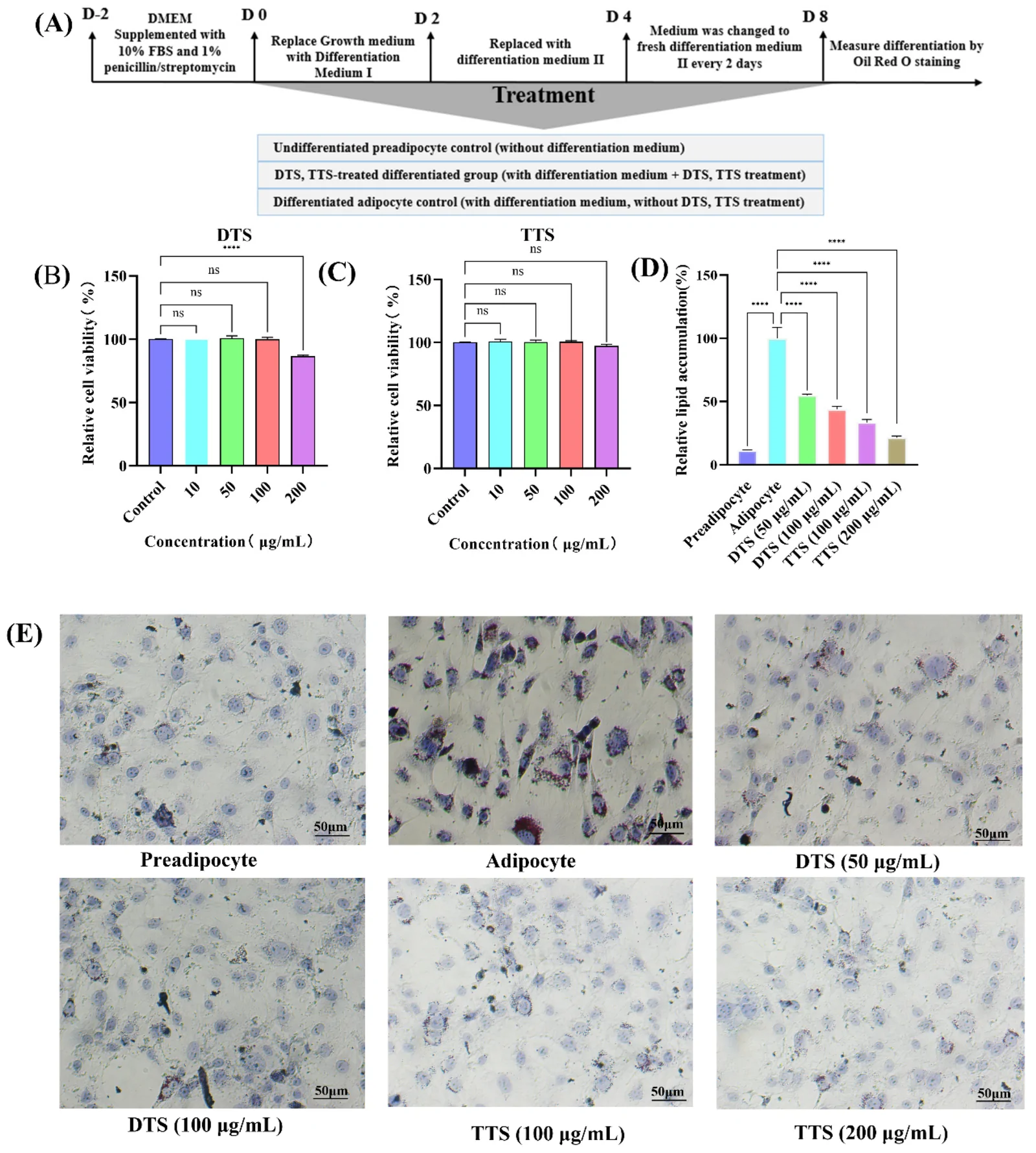
Effect of DTS and TTS on the differentiation of 3T3-L1 preadipocytes. (**A**) Schematic diagram of the 3T3-L1 adipocyte differentiation protocol; D−2, D0, D2, D4, and D8 indicate days −2, 0, 2, 4, and 8 relative to treatment, respectively. (**B**) cell viability of 3T3-L1 preadipocytes treated with DTS at concentrations of 10, 50, 100, and 200 μg/mL. (**C**) cell viability of differentiated 3T3-L1 adipocytes treated with TTS at concentrations of 10, 50, 100, and 200 μg/mL. (**D**) quantification of relative lipid accumulation by Oil Red O extraction in differentiated adipocytes treated with DTS (50 and 100 μg/mL) and TTS (100 and 200 μg/mL). (**E**) Oil Red O staining of lipid droplets (magnification, 200×, scale bar: 50 μm) in differentiated adipocytes treated with DTS (50 and 100 μg/mL) and TTS (100 and 200 μg/mL). Preadipocyte (undifferentiated) and Adipocyte (MDI-induced) serve as controls. Data are presented as means ± standard error of the mean (SEM) (*n* = 3). ns, not significant (*p* > 0.05), **** *p* < 0.001 vs. respective control group in (**B**); **** *p* < 0.001 vs. adipocytes in (**D**).

The experimental protocol for 3T3-L1 adipocyte differentiation and the corresponding treatment groups are illustrated in Figure 2A. Briefly, preadipocytes were induced to differentiate over 8 days with or without saponin extracts, and the effects were evaluated at multiple endpoints. The cytotoxic effects of DTS and TTS on 3T3-L1 preadipocytes were first assessed using the MTT assay. Cells were treated with DTS or TTS at concentrations of 10, 50, 100, and 200 μg/mL for 48 h. As shown in Figure 2B,C, both extracts exhibited minimal cytotoxicity at lower concentrations. Notably, only DTS at the highest concentration (200 μg/mL) showed a slight inhibitory effect on cell viability, with a survival rate of 86.5%, while TTS maintained high viability at the same concentration. Based on these results, concentrations of 50 and 100 μg/mL were selected for DTS, and concentrations of 100 and 200 μg/mL were selected for TTS for subsequent differentiation experiments, as these concentrations exhibited negligible cytotoxicity.

To quantify the inhibitory effects on adipogenesis, Oil Red O staining was performed, and the extracted dye was measured spectrophotometrically (Figure 2D). Compared to the differentiated control group, treatment with either DTS or TTS significantly reduced lipid accumulation in a dose-dependent manner, with the high-dose groups showing greater inhibition. Because the two extracts were tested over different concentration ranges (50–100 μg/mL for DTS and 100–200 μg/mL for TTS), their relative inhibitory potency could not be directly compared.

Microscopic examination (200×) of Oil Red O-stained cells (Figure 2E) revealed abundant and densely distributed red-stained lipid droplets in the differentiated control group compared to the undifferentiated preadipocyte control, confirming successful differentiation. Treatment with DTS or TTS markedly reduced both the number and size of lipid droplets in a dose-dependent manner, with the high-dose groups exhibiting more pronounced effects than the low-dose groups.

### 3.3. Effects of DTS and TTS on Adipogenesis and Fatty Acid Oxidation-Related Gene and Protein Expression in 3T3-L1 Adipocytes

**Figure 3:**
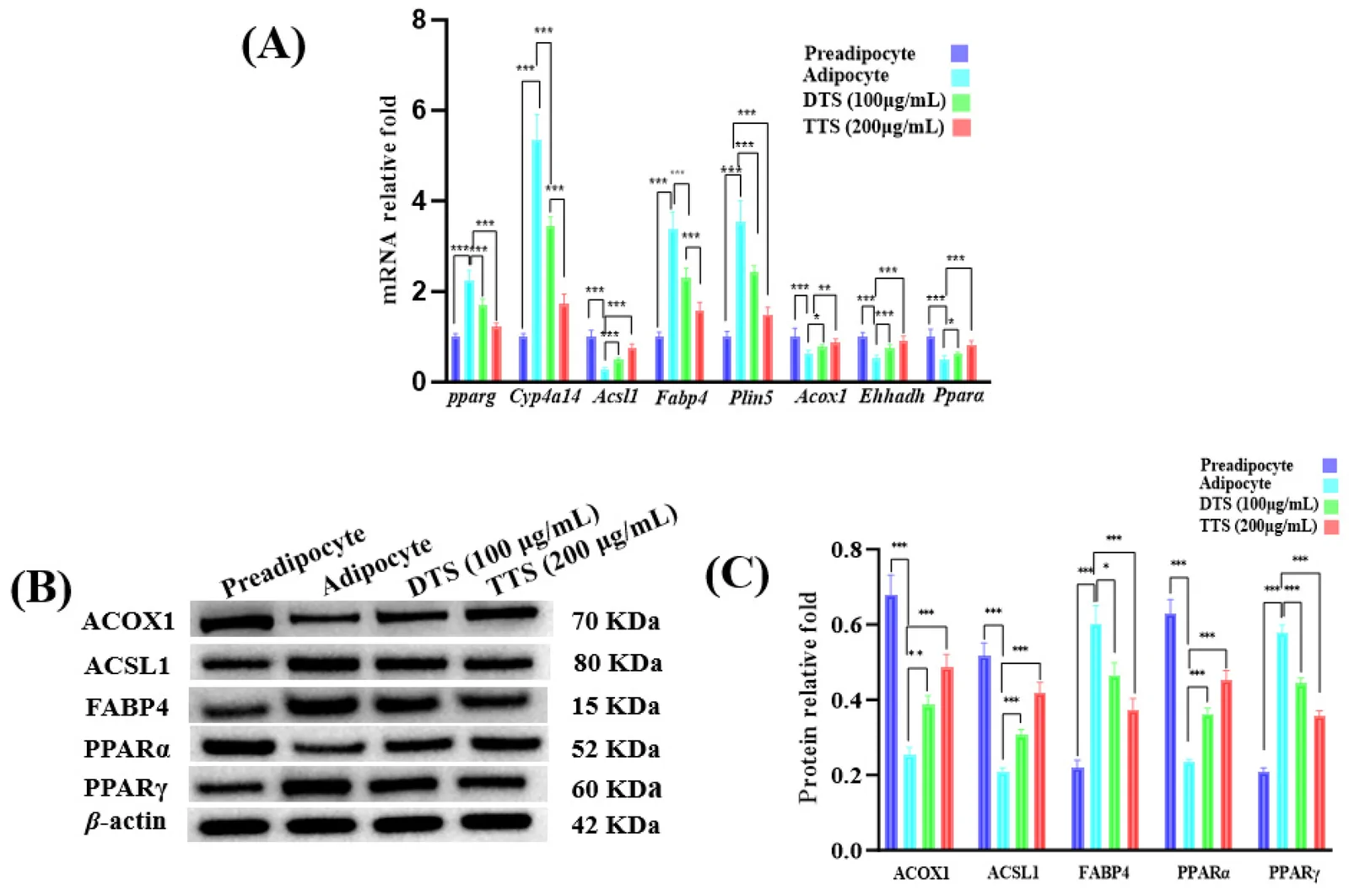
Effects of DTS and TTS on the expression of lipid metabolism-related genes and proteins in differentiated adipocytes. (**A**) Relative mRNA expression levels of Pparg, Cyp4a14, Acsl1, Fabp4, Plin5, Acox1, Ehhadh, and Ppara, normalized to GAPDH as an internal control. (**B**) Representative Western blots showing protein expression of ACOX1, ACSL1, FABP4, PPARα, and PPARγ, with β-actin as a loading control. (**C**) Quantitative analysis of protein expression normalized to β-actin. Data are presented as mean ± standard error of the mean (SEM), with *n* = 3 independent experiments per group. * *p* < 0.05, ** *p* < 0.01, *** *p* < 0.001 vs. differentiated adipocytes (Adipocyte group).

To elucidate the molecular underpinnings of the observed anti-adipogenic and lipid-lowering effects of DTS and TTS, we examined the expression of pivotal genes and proteins governing adipocyte differentiation and fatty acid metabolism. As shown in Figure 3A, differentiation robustly upregulated the mRNA levels of the adipogenic master transcription factor *Pparg* and its downstream target *Fabp4*, as well as *Plin5* and *Cyp4a14*, which are associated with lipid droplet dynamics and fatty acid ω-oxidation. Concurrently, a marked suppression of *Acsl1*, *Pparα*, *Acox1*, and *Ehhadh* transcripts, which are critical for fatty acid activation and *β*-oxidation, was observed in differentiated adipocytes. Treatment with DTS (100 μg/mL) or TTS (200 μg/mL) effectively counteracted these differentiation-associated alterations: both extracts significantly attenuated the expression of *Pparg*, *Fabp4*, *Plin5*, and *Cyp4a14*, while substantially restoring the mRNA levels of *Acsl1*, *Pparα*, *Acox1*, and *Ehhadh*. Notably, TTS exhibited a more pronounced inhibitory effect on adipogenic gene expression, reducing Pparg to near-Control levels and more robustly downregulating *Fabp4*, *Plin5*, and *Cyp4a14* compared with DTS.

Consistent with the transcriptional data, Western blot analysis demonstrated that DTS and TTS modulate key adipogenic and oxidative pathways at the protein level (Figure 3B,C). While differentiated adipocytes exhibited markedly elevated protein levels of PPARγ and FABP4 alongside reduced expression of ACSL1, PPARα, and ACOX1, treatment with either DTS or TTS effectively reversed these changes. Both extracts significantly upregulated ACSL1, PPARα, and ACOX1 protein expression, indicating enhanced fatty acid oxidative capacity, while concurrently suppressing PPARγ and FABP4 expression, reflecting inhibited adipogenic commitment. Collectively, these results indicate that DTS and TTS shifted adipocyte metabolism toward a pattern consistent with dual modulation of the two signaling axes: the anti-adipogenic effect was accompanied by downregulation of the PPARγ/FABP4 cascade, and the enhanced lipid catabolism was accompanied by upregulation of the PPARα/ACOX1-mediated β-oxidation pathway. The coordinated regulation of these two pathways provides a mechanistic basis for the potent anti-adipogenic and lipid-reducing activities of the triterpenoid saponin extracts from *V. angularis*.

### 3.4. DTS and TTS Attenuate HFD-Induced Obesity, Dyslipidemia, Hepatic Steatosis, and Adipose Tissue Remodeling in Mice

**Figure 4:**
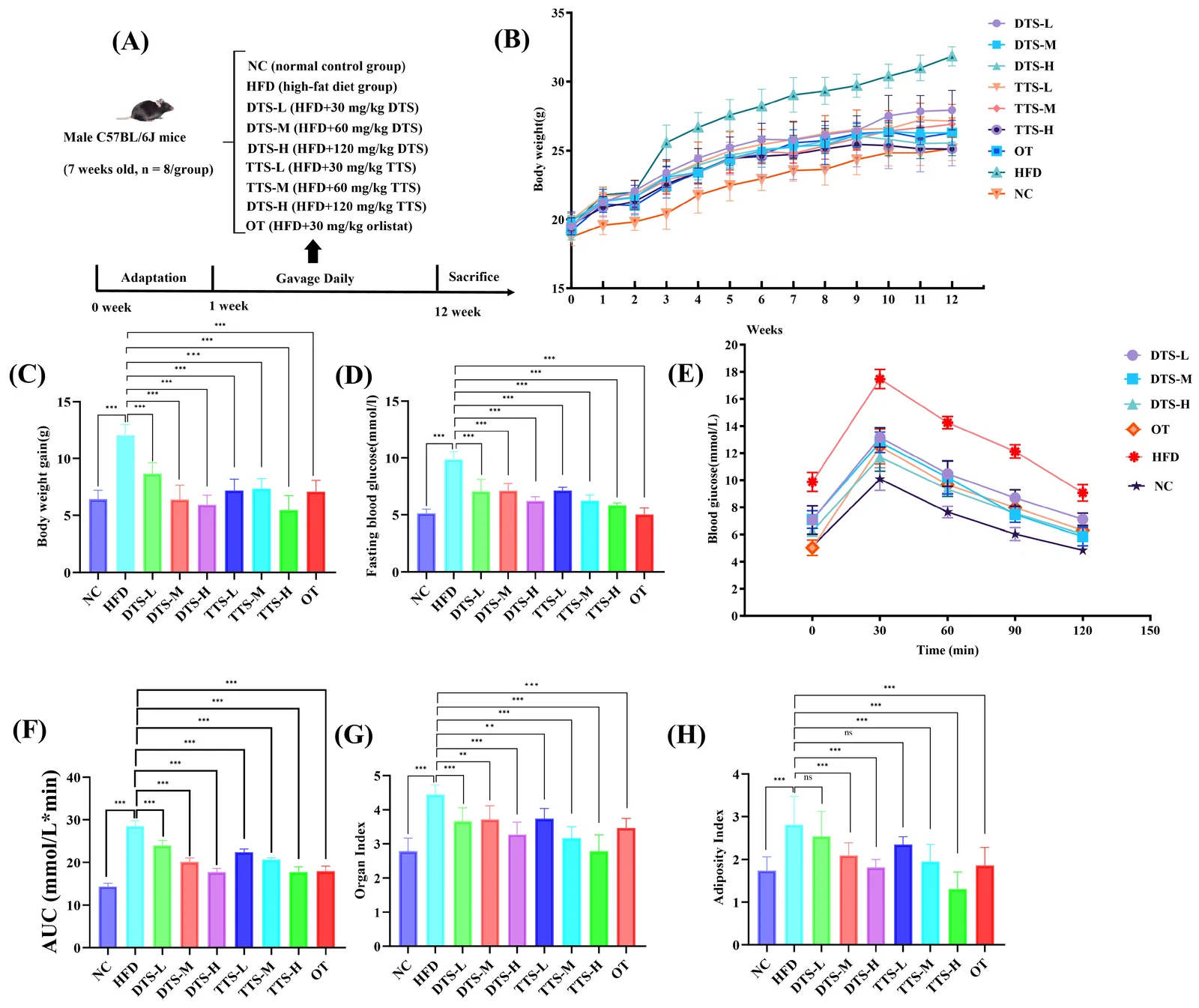
Effects of DTS and TTS on body weight gain, adiposity index, organ index and glucose tolerance in HFD-fed mice. (**A**) Schematic diagram of the entire animal testing process. (**B**) Body weight changes over a 12-week feeding period; (**C**) T = total body weight gain at the end of the experiment. (**D**) Fasting blood glucose levels after 12 weeks of intervention; (**E**) blood glucose levels in the oral glucose tolerance test (OGTT). Mice were fed a normal diet (NC), high-fat–high-cholesterol experimental diet (HFD), or HFD supplemented with orlistat (OT) as a positive control or DTS and TTS at low (DTS and TTS-L), medium (DTS and TTS-M), and high (DTS and TTS-H) doses. (**F**) Area under the curve (AUC) of blood glucose levels. (**G**) Organ index (organ weight/body weight × 100%) calculated for liver. (**H**) Adiposity index (total white adipose tissue weight/body weight × 100%). Data are expressed as mean ± standard error of the mean (SEM), *n* = 8 per group. ns, not significant (*p* > 0.05), ** *p* < 0.01, *** *p* < 0.001 vs. HFD group.

To systematically evaluate the in vivo anti-obesity efficacy of DTS and TTS, body weight was monitored weekly throughout the 12-week intervention period (Figure 4A). As shown in Figure 4B, from the third week onward, all treatment groups exhibited markedly slower weight gain compared with the HFD-fed Model group (*p* < 0.01 vs. Model for all treated groups). At the end of the experiment, DTS and TTS dose-dependently reduced body weight gain, with the high-dose groups showing the most pronounced effects—achieving reductions generally comparable to those of OT (Figure 4C), with TTS-H showing a significantly greater reduction than OT (*p* < 0.05, Tukey’s HSD). Notably, TTS-H demonstrated the greatest weight-lowering efficacy among all tested extracts, with its effect reaching statistical significance versus both Model and several lower-dose groups.

Given the close association between obesity and insulin resistance, an oral glucose tolerance test (OGTT) was performed three days prior to sacrifice. As shown in Figure 4D, fasting blood glucose levels were significantly elevated in the Model group compared with the Control group (*p* < 0.01), confirming the development of insulin resistance in HFD-fed mice. Following glucose administration, blood glucose peaked at 30 min in all groups. At 60 min, the Control group exhibited a marked decline (*p* < 0.01 vs. Model), whereas the Model group maintained persistently elevated levels. Notably, all treatment groups and the OT group showed significantly reduced blood glucose at 120 min (*p* < 0.05 vs. Model for each) (Figure 4E). The area under the curve (AUC) analysis (Figure 4F) further demonstrated that the Model group had a significantly higher AUC than the Control group (*p* < 0.01), while all treatment groups exhibited significantly reduced AUC values compared with the Model group (*p* < 0.01 for each), indicating improved glucose tolerance.

Chronic HFD consumption promotes ectopic lipid deposition in adipose depots and the liver. As depicted in Figure 4G,H, the Model group exhibited significantly increased adipose and liver coefficients compared with the Control group (*p* < 0.01). The adipose coefficient was significantly reduced in the DTS-M, DTS-H, TTS-M, TTS-H, and OT groups relative to the Model group (*p* < 0.05–0.001, Dunnett’s test), with DTS-H and TTS-H demonstrating the most substantial reductions. Additionally, liver coefficients were markedly decreased in the DTS-H, TTS-M, TTS-H, and OT groups (*p* < 0.01 vs. Model for each), confirming the inhibitory effects of these extracts on hepatic fat accumulation.

**Figure 5:**
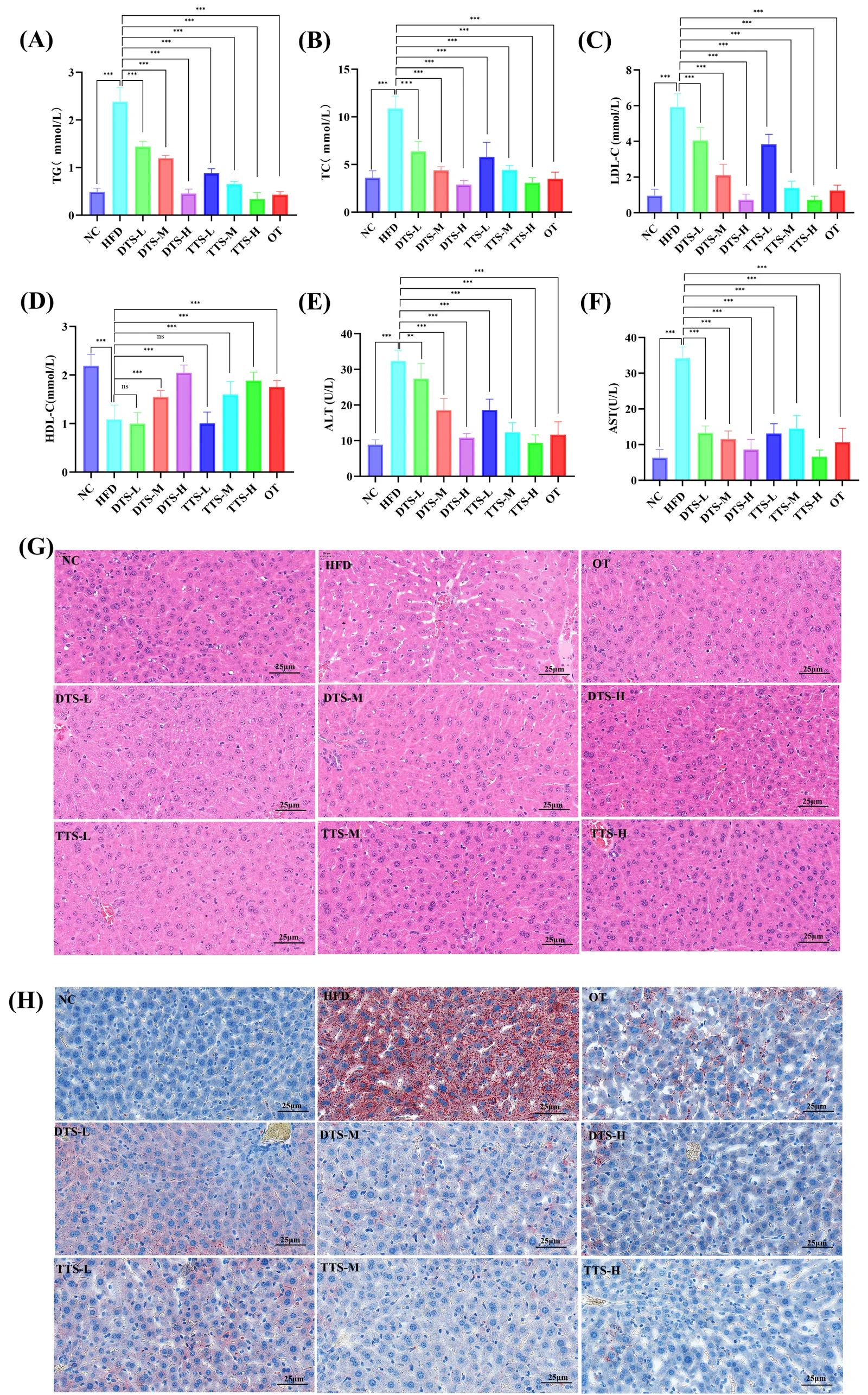
Effects of DTS and TTS on serum lipid profiles and liver function markers in HFD-fed mice. (**A**) Triglyceride (TG); (**B**) total cholesterol (TC); (**C**) low-density lipoprotein cholesterol (LDL-C); (**D**) high-density lipoprotein cholesterol (HDL-C); (**E**) alanine aminotransferase (ALT); and (**F**) aspartate aminotransferase (AST) levels in serum. (**G**) Hematoxylin and eosin (H&E) staining of liver (40× magnification, 25 μm); (**H**) Oil Red O staining of liver (40× magnification, 25 μm). ns, not significant (*p* > 0.05), ** *p* < 0.01 vs. HFD group, *** *p* < 0.001 vs. HFD group.

Obesity is frequently accompanied by dyslipidemia and hepatic dysfunction. As shown in Figure 5A–D, HFD feeding significantly elevated serum triglyceride (TG), total cholesterol (TC), and low-density lipoprotein cholesterol (LDL-C) levels, while simultaneously reducing high-density lipoprotein cholesterol (HDL-C) compared with the Control group (*p* < 0.01). Treatment with DTS and TTS markedly reversed these lipid abnormalities, with significant reductions in TG, TC, and LDL-C (*p* < 0.01 vs. Model for each dose) and significant increases in HDL-C (*p* < 0.01 vs. Model for high doses). Serum alanine aminotransferase (ALT) and aspartate aminotransferase (AST) activities, key biomarkers of hepatic injury, were markedly elevated in the Model group relative to the Control group (*p* < 0.01). Both OT and all DTS and TTS treatment groups significantly reduced ALT and AST levels (*p* < 0.01 vs. Model), indicating potent hepatoprotective effects (Figure 5E,F). Direct pairwise comparisons between DTS and TTS at matched dose levels (Tukey’s HSD) are provided in Table S6.

In brown adipose tissue (BAT), the Control group displayed typical mature BAT morphology characterized by small, densely packed multilocular adipocytes with numerous small lipid droplets, centrally located round nuclei, and abundant capillaries and interstitium (Figure S2A). Conversely, the Model group exhibited profound BAT “whitening,” with enlarged unilocular adipocytes displaying large fused lipid droplets, peripherally flattened nuclei, markedly loose cellular arrangement, and significantly reduced capillary density—closely resembling WAT morphology (*p* < 0.01 vs. Control). Treatment with DTS, TTS, or OT dose-dependently reversed these whitening changes, as evidenced by reduced adipocyte size, decreased lipid droplet fusion, and restoration of multilocular morphology (*p* < 0.01 vs. Model for high-dose groups), indicating preservation of BAT thermogenic function.

Histopathological examination of white adipose tissue (WAT) revealed that the Control group exhibited small, tightly packed, and uniformly sized adipocytes, whereas the Model group displayed markedly enlarged, loosely arranged adipocytes with ill-defined boundaries (Figure S2B). Treatment with DTS and TTS dose-dependently reversed these morphological abnormalities, restoring tighter cellular organization and reducing adipocyte size (*p* < 0.01 vs. Model for all treated groups), thereby confirming the inhibitory effects of these extracts on HFD-induced adipose tissue expansion and hypertrophy.

Collectively, these findings demonstrate that DTS and TTS alleviate HFD-induced obesity, dyslipidemia, hepatic steatosis, insulin resistance, and adipose tissue remodeling in this mouse model in a dose-dependent manner. The high-dose regimens of DTS and TTS achieved overall efficacy comparable to that of the positive control orlistat, supporting their further evaluation as candidate natural anti-obesity agents.

### 3.5. Transcriptomic Profiling Reveals Distinct Hepatic Metabolic Reprogramming by DTS and TTS in HFD-Induced Obese Mice

**Figure 6:**
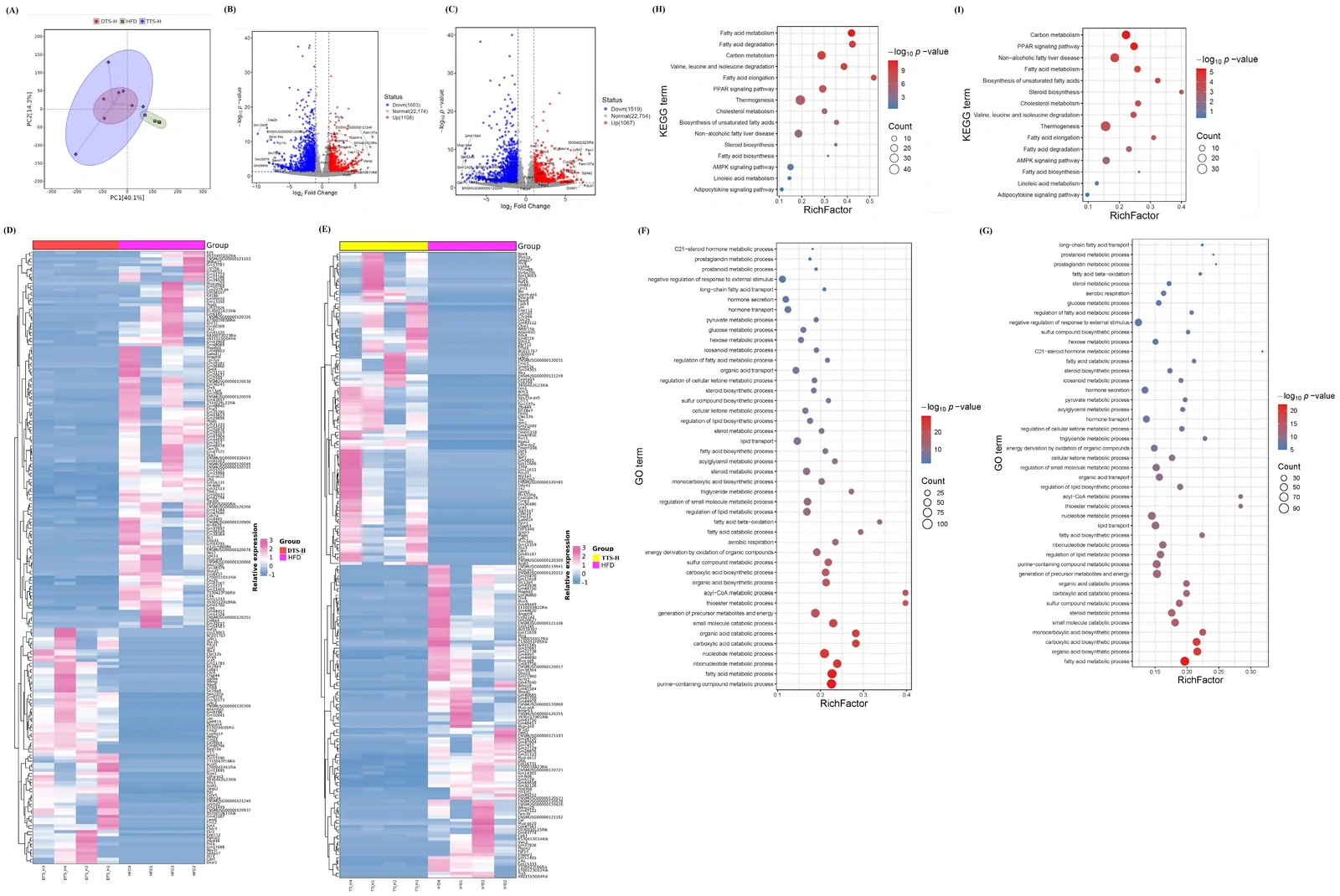
Transcriptomic profiling of liver tissues from HFD-fed mice treated with DTS or TTS. (**A**) Principal component analysis (PCA) plot showing separation of samples across three groups: HFD (cyan), DTS-H (magenta), and TTS-H (orange). (**B**,**C**) Volcano plots displaying DEGs with significance (−log_10_ *p*-value vs. log_2_ fold change) for DTS-H vs. HFD (**B**) and TTS-H vs. HFD (**C**); blue dots indicate downregulated genes, red dots upregulated genes, and gray dots non-significant changes. (**D**,**E**) Heatmaps of differentially expressed genes (DEGs) based on hierarchical clustering, showing gene expression patterns between HFD group and DTS-H group (**D**), and between HFD group and TTS-H group (**E**); color scale represents relative expression levels (blue: low; red: high). (**F**,**G**) Gene Ontology (GO) enrichment analysis of DEGs, illustrating enriched biological processes (BP), cellular components (CC), and molecular functions (MF) based on −log_10_ *p*-value and rich factor for DTS-H vs. HFD (**F**) and TTS-H vs. HFD (**G**); (**H**,**I**) KEGG pathway enrichment analysis highlighting significantly enriched metabolic pathways for DTS-H vs. HFD (**H**) and TTS-H vs. HFD (**I**), with dot size representing gene count and color intensity indicating −log_10_ *p*-value.

To systematically investigate the molecular mechanisms underlying the hepatoprotective and lipid-lowering effects of DTS and TTS, transcriptome profiling was performed on liver tissues from HFD-fed obese mice treated with the most effective doses, namely DTS-H and TTS-H, in comparison with the Model group. Principal component analysis (PCA) revealed distinct separations among the HFD, DTS-H, and TTS-H groups, indicating clear global transcriptomic differences induced by the two treatments (Figure 6A). Differential expression analysis, using DESeq2 with criteria of |log_2_(Fold Change)| > 1 and adjusted *p*-value ≤ 0.05, identified 2711 differentially expressed genes (DEGs) in the DTS-H group compared with the Model group, comprising 1108 upregulated and 1603 downregulated genes. In the TTS-H group, 2586 DEGs were identified, with 1067 upregulated and 1519 downregulated genes. The DEG distributions were visualized using volcano plots (Figure 6B,C), in which red and blue dots represent significantly upregulated and downregulated genes, respectively. Hierarchical clustering heatmaps further illustrated the distinct expression patterns of these DEGs between the HFD and DTS-H groups, as well as between the HFD and TTS-H groups (Figure 6D,E).

To compare the functional impacts of DTS and TTS interventions on hepatic metabolism, Gene Ontology (GO) enrichment analysis was performed for both DTS-H vs. Model and TTS-H vs. Model comparisons (Figure 6F,G). Both treatments significantly enriched biological processes related to fatty acid metabolism, fatty acid β-oxidation, triglyceride metabolism, and organic acid catabolism, indicating that both extracts promote hepatic fatty acid utilization and reduce lipid accumulation. Additionally, enrichment of aerobic respiration and energy production pathways suggested enhanced mitochondrial function and increased energy expenditure. Steroid metabolism and its biosynthetic processes were also modulated, implying an impact on cholesterol/sterol homeostasis. Notably, the TTS-H group exhibited broader regulatory effects on glycolipid metabolism, with additional enrichment of hexokinase/glucokinase activity, regulation of lipid biosynthesis, peptide hormone secretion, and circadian rhythm pathways. These findings suggest that TTS engages a broader range of obesity-associated multi-system metabolic pathways, simultaneously improving glucose metabolism, restraining excessive lipid synthesis, and modulating metabolic hormones and inflammatory pathways.

Kyoto Encyclopedia of Genes and Genomes (KEGG) pathway enrichment analysis further substantiated these observations (Figure 6H,I). Compared with the Model group, the DTS-H group showed the most prominently enriched pathways in thermogenesis, carbon metabolism, and non-alcoholic fatty liver disease (NAFLD). In contrast, the TTS-H group exhibited significant enrichment in the PPAR signaling pathway, biosynthesis of unsaturated fatty acids, fatty acid metabolism, fatty acid elongation, and fatty acid degradation. Both DTS and TTS upregulated key components of the PPAR signaling axis, accompanied by increased hepatic expression of fatty acid β-oxidation-related genes, enhanced thermogenic capacity, and ameliorated NAFLD-associated pathologies. Despite these shared features, distinct differences in pathway engagement were observed: DTS-H enriched a greater number of genes within fatty acid catabolic pathways, suggesting a more potent pro-lipolytic effect, whereas TTS-H uniquely and significantly enriched the peroxisome pathway, implicating peroxisomal β-oxidation as an additional mechanism of action.

Collectively, these transcriptomic analyses reveal that both DTS and TTS effectively ameliorate hepatic lipid metabolic disorders in obese mice, yet they operate through distinct yet complementary mechanisms. DTS exhibits the strongest pro-lipolytic and thermogenic effects, while TTS preferentially engages PPAR-mediated fatty acid metabolism and elongation, coupled with activation of peroxisomal oxidation pathways. This divergence in mechanistic hierarchy provides a molecular basis for the differential metabolic benefits conferred by these two triterpenoid saponin extracts.

### 3.6. DTS and TTS Modulate Hepatic Lipid Metabolism Involving Dual Regulation of PPARγ/Fabp4 and PPARα/Acox1 Signaling Axes

**Figure 7:**
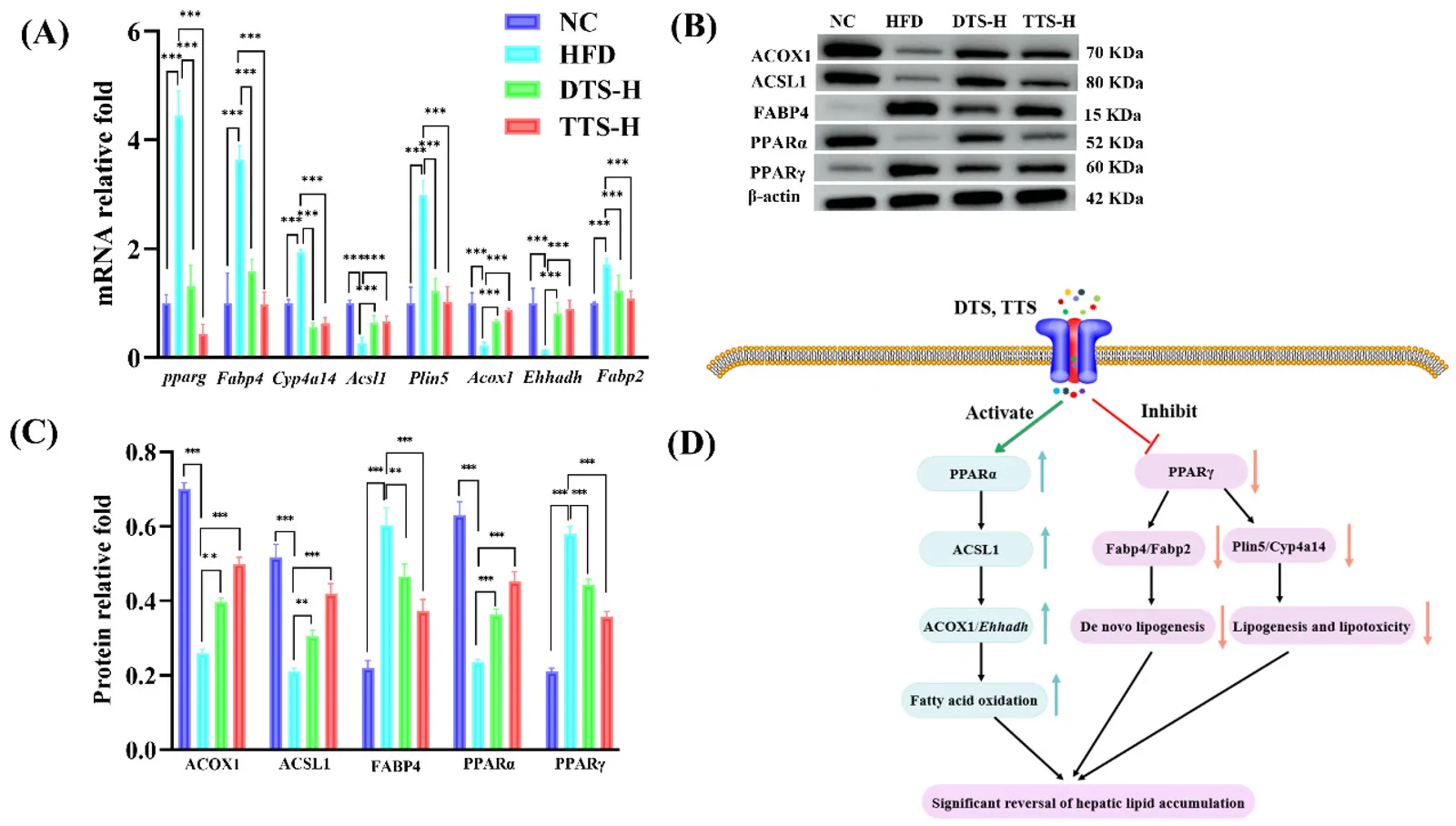
Effects of DTS and TTS on hepatic lipid metabolism-related gene and protein expression in liver tissues of HFD-fed mice. (**A**) mRNA expression levels of *Pparg*, *Fabp4*, *Fabp2*, *Plin5*, *Cyp4a14*, *Acsl1*, *Acox1*, and *Ehhadh* were determined by qRT-PCR and normalized to Gapdh. (**B**) Representative Western blot images showing protein levels of ACOX1, ACSL1, FABP4, PPARα, and PPARγ with β-actin as the loading control. (**C**) Quantification of protein expression. (**D**) Schematic of the representative mechanisms for the action of DTS and TTS on obesity. Black solid arrows indicate the direction of the signaling pathway or promoting effects. Green arrows labeled “Activate” indicate activation. Red blunt-ended lines labeled “Inhibit” indicate inhibition. Light blue upward arrows indicate upregulation or increased expression/activity. Orange downward arrows indicate downregulation or decreased expression/activity. ** *p* < 0.01, *** *p* < 0.001 vs. the HFD group.

To elucidate the molecular mechanisms underlying the hepatoprotective and lipid-lowering effects of DTS and TTS, we examined the mRNA expression of key genes governing hepatic lipogenesis, lipid droplet dynamics, and fatty acid oxidation using quantitative real-time PCR. As shown in Figure 7A, HFD feeding induced profound hepatic metabolic dysfunction, characterized by marked upregulation of the adipogenic master transcription factor *Pparg*, its downstream effectors *Fabp4* and *Fabp2*, as well as *Plin5* (associated with lipid droplet formation) and *Cyp4a14* (involved in fatty acid ω-oxidation) (*p* < 0.01 vs. Control). Concurrently, the expression of *Acsl1* (which activates long-chain fatty acids for β-oxidation), *Acox1* (a key peroxisomal β-oxidation enzyme), and *Ehhadh* (another peroxisomal oxidation gene) was drastically suppressed (*p* < 0.01 vs. Control), indicating a severe impairment of hepatic oxidative capacity. These transcriptional abnormalities collectively confirmed the successful establishment of a hepatic lipid metabolic disorder model in HFD-fed obese mice.

Both DTS-H and TTS-H profoundly reversed these HFD-induced alterations. Both extracts effectively suppressed the overexpression of *Pparg*, *Fabp4*, *Fabp2*, *Plin5*, and *Cyp4a14* (all *p* < 0.01 vs. Model), thereby inhibiting the lipogenic programme and reducing lipid droplet accumulation. In parallel, both DTS-H and TTS-H significantly restored the mRNA levels of *Acsl1* and *Ehhadh* (*p* < 0.01 vs. Model). DTS-H also upregulated Acox1 (*p* < 0.05 vs. Model), whereas TTS-H upregulated *Acox1* to a greater extent (*p* < 0.01 vs. Model), indicating enhanced recovery of fatty acid oxidative capacity.

Notably, direct pairwise comparison (Tukey’s HSD) revealed that TTS-H suppressed *Pparg* and *Fabp4* to a significantly greater extent than DTS-H (*p* < 0.001 and *p* < 0.01, respectively) and restored *Acox1* mRNA to a significantly higher level (*p* < 0.001). However, the two extracts did not differ significantly on the remaining five genes (all *p* > 0.05). Taken together, these findings indicate that both DTS-H and TTS-H ameliorate hepatic lipid metabolic disorders through dual regulation of lipogenesis and fatty acid oxidation, with TTS-H showing significantly stronger effects on *Pparg*, *Fabp4*, and *Acox1*.

To further validate the molecular mechanisms, we assessed the protein expression of key components of the PPARγ/FABP4 and PPARα/ACOX1 axes via Western blotting (Figure 7B,C). HFD feeding in the Model group markedly elevated the protein levels of PPARγ and its downstream target FABP4 compared with the Control group (*p* < 0.001), confirming that long-term HFD activates hepatic adipogenic pathways, driving lipid synthesis and aberrant accumulation. Remarkably, treatment with either DTS-H or TTS-H profoundly reversed these abnormalities, significantly downregulating PPARγ and FABP4 protein expression (*p* < 0.001 vs. Model). These results indicate that the reduction in hepatic lipogenic markers was accompanied by downregulation of the PPARγ/FABP4 axis.

Concurrently, the protein levels of PPARα and its downstream fatty acid metabolic enzymes ACSL1 and ACOX1 were significantly reduced in the Model group compared with the Control group (*p* < 0.001), suggesting that HFD profoundly suppresses hepatic fatty acid activation and β-oxidation, thereby exacerbating lipid accumulation. Treatment with DTS-H or TTS-H significantly restored the protein expression of PPARα, ACSL1, and ACOX1 (*p* < 0.001 vs. Model), consistent with engagement of the PPARα-mediated fatty acid oxidation pathway and enhanced fatty acid catabolic capacity.

Collectively, these results demonstrate that DTS and TTS ameliorate hepatic lipid metabolism disorders through coordinated multi-target regulation at both transcriptional and translational levels. In both extracts, the suppression of lipogenic markers coincided with downregulation of the PPARγ/FABP4 axis, while concurrently promoting fatty acid oxidation coincided with upregulation of the PPARα/ACSL1/ACOX1 pathway (Figure 7D). Notably, TTS-H exerted significantly stronger regulation than DTS-H on a subset of molecular endpoints—the lipogenic genes Pparg and Fabp4, the fatty acid oxidation gene Acox1, and all five proteins examined (*p* < 0.05–0.001, Tukey’s HSD)—whereas the two extracts were statistically indistinguishable on the remaining genes. This dual regulatory mechanism—inhibiting lipid synthesis while enhancing oxidative catabolism—underpins the potent hepatoprotective and lipid-lowering effects of DTS and TTS in obesity.

### 3.7. DTS and TTS Counteract HFD-Induced Gut Dysbiosis via Distinct Microbiota Modulation Profiles

**Figure 8:**
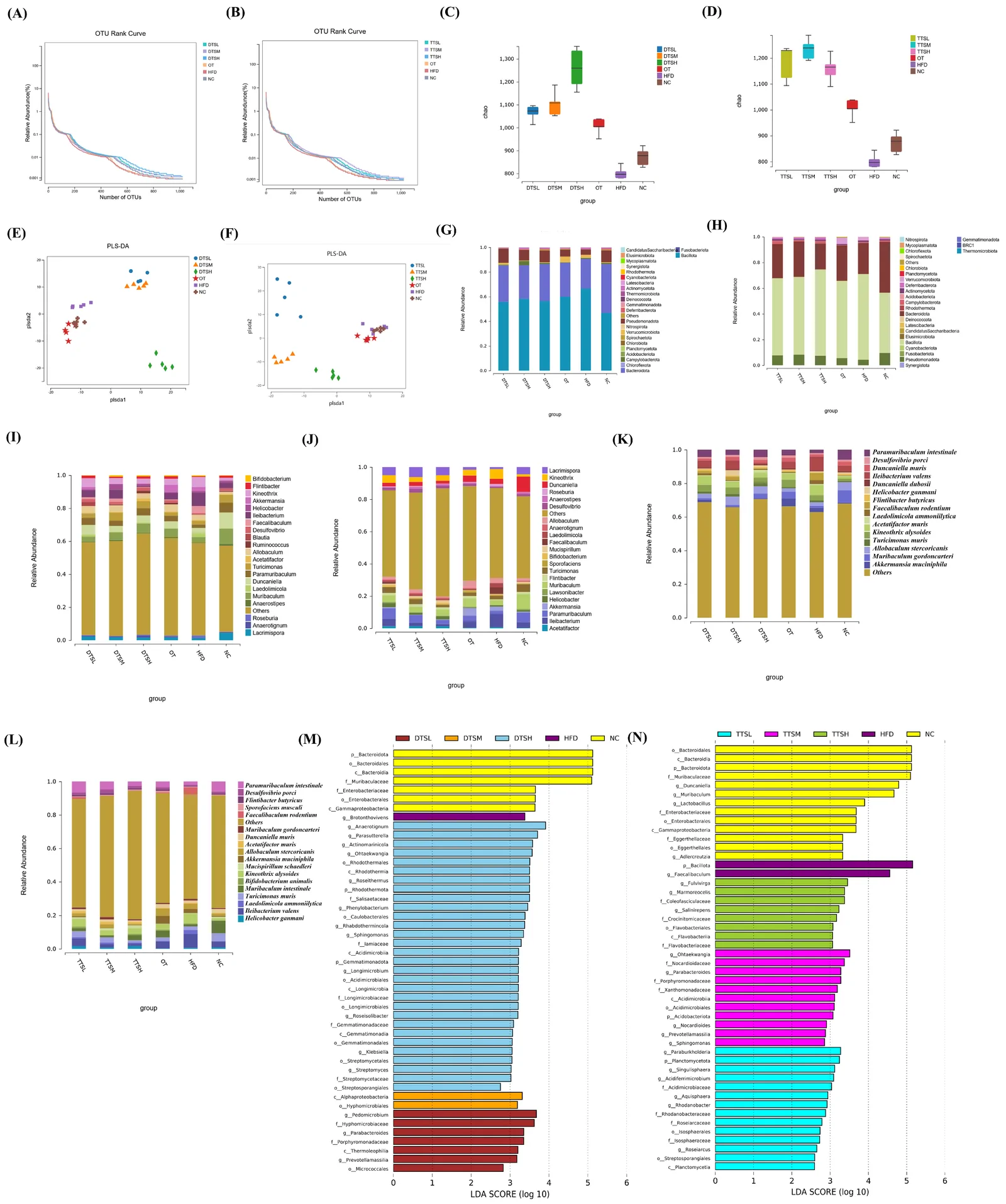
DTS and TTS remodel the gut microbiota composition in HFD-fed mice. (**A**,**B**) OTU rank-abundance curves reflecting the species richness and evenness across different groups. (**C**,**D**) Boxplots showing the Chao1 index of alpha diversity. (**C**) DTS groups; (**D**) TTS groups. (**E,F**) PLS-DA score plots showing the separation of the DTS (**E**) and TTS (**F**) groups based on the first two PLS-DA components. (**G**,**H**) Gut microbiota composition at the phylum level. (**I**,**J**) Gut microbiota composition at the genus level. (**K**,**L**) Gut microbiota composition at the species level. (**M**,**N**) LEfSe analysis showing the LDA scores of differentially abundant bacterial taxa (LDA > 2, *p* < 0.05) among different groups.

To investigate the role of gut microbiota in the anti-obesity effects of DTS and TTS, we performed 16S rRNA gene sequencing on cecal samples from mice across all experimental groups. As shown in the rank-abundance curves plotted separately for the DTS (Figure 8A) and TTS (Figure 8B) groups, HFD feeding substantially reduced microbial evenness. Notably, DTS-H and TTS-H interventions both effectively restored the species distribution equilibrium toward the normal control profile. Alpha diversity analysis based on the Chao1 index (Figure 8C,D) revealed that HFD feeding significantly decreased gut microbial richness. Notably, all six intervention groups (TTS-L, TTS-M, TTS-H, DTS-L, DTS-M, and DTS-H) exhibited Chao1 indices that markedly exceeded the normal control (NC) baseline. Collectively, these observations indicate that both TTS and DTS treatments, irrespective of the applied intervention level, potently augmented gut microbial richness beyond the physiological homeostasis by promoting the expansion of rare bacterial taxa. Beta diversity analysis via PLS-DA (Figure 8E,F) revealed distinct structural alterations induced by the interventions. In the DTS analysis (Figure 8E), the NC and HFD groups exhibited clear segregation, substantiating the profound impact of the high-fat diet on microbial community assembly. Spatially, DTS-L and DTS-M were predominantly positioned in the upper-right quadrant, while DTS-H diverged to the lower-right region. In comparison, in the TTS analysis (Figure 8F), the NC and HFD groups displayed a relatively limited separation, potentially attributable to biological variability inherent to the HFD-induced gut microbial landscape. Nevertheless, all TTS intervention groups formed distinct, independent clusters located far from both baseline groups, with TTS-L specifically occupying the upper-left quadrant and TTS-M along with TTS-H diverging toward the lower-left region. Collectively, these distinct topological patterns suggest that both DTS and TTS treatments actively induced targeted, intervention-specific compositional remodeling rather than restoring the microbiota to the NC status. Additionally, the OT group consistently clustered in close proximity to the HFD group in both PLS-DA plots.

At the phylum level, Bacillota (formerly Firmicutes) and Bacteroidota (formerly Bacteroidetes) constituted the predominant phyla across all experimental groups (Figure 8G,H, Tables S7 and S8). HFD feeding markedly elevated the Firmicutes/Bacteroidetes (F/B) ratio relative to the NC group; given the current controversy surrounding the F/B ratio as a single marker of metabolic health, this ratio is reported here only as a phylum-level compositional descriptor and is not interpreted in isolation. DTS intervention (DTS-L, DTS-M, and DTS-H) reduced the relative abundance of Bacillota and increased that of Bacteroidota, shifting the F/B ratio toward the NC baseline (Figure 8G). TTS intervention produced a non-linear, concentration-dependent phylum-level reconfiguration (Figure 8H): TTS-L and TTS-M shifted the F/B ratio modestly downward relative to the HFD group, whereas TTS-H further elevated Bacillota and reduced Bacteroidota, yielding an F/B ratio above the HFD baseline. The divergent phylum-level trajectories of DTS and TTS, rather than supporting simple “normalization” of microbial communities, highlight that the two extracts engage structurally distinct compositional programs—an observation that is more clearly resolved at the genus level (see below). Meanwhile, the OT group consistently maintained an intermediate F/B ratio between the HFD and NC groups.

At the genus level (Figure 8I,J, Tables S9 and S10), HFD feeding profoundly perturbed the gut microbiota composition compared with the NC group. The DTS and TTS interventions induced distinct genus-specific modulatory profiles, characterized by marked concentration-dependent responsiveness. Specifically, the relative abundance of *Bifidobacterium* was significantly enriched in the DTS-M and TTS-L groups. Both DTS and TTS extracts consistently exerted potent suppression against the obesity-associated genera *Faecalibaculum* and *Ileibacterium*, with DTS-H, TTS-M, and TTS-H demonstrating particularly pronounced inhibitory activities. Moreover, while TTS-L and TTS-M partially reversed the depletion of *Paramuribaculum*, DTS-M and DTS-H effectively reinstated the abundance of *Turicimonas*. Collectively, DTSH manifested the most comprehensive genus-level restructuring in terms of suppressing pathobionts and replenishing beneficial commensals, whereas TTSL conferred the most notable *Bifidobacterium*-enriching capacity.

At the species level (Figure 8K,L, Tables S11 and S12), the HFD challenge profoundly perturbed the gut microbial equilibrium, characterized by a marked enrichment of *Ileibacterium valens*, *Faecalibaculum rodentium*, and *Akkermansia muciniphila*, concomitant with a substantial depletion of beneficial commensals such as *Muribaculum intestinale*, *Paramuribaculum intestinale*, and *Turicimonas muris*. The DTS intervention substantially counteracted these HFD-induced perturbations (Figure 8K), eliciting a conspicuous suppression of the pro-inflammatory species *Ileibacterium valens* and *Faecalibaculum rodentium*, while concurrently replenishing *Muribaculum intestinale*, *Paramuribaculum intestinale*, and *Turicimonas muris*. Furthermore, DTS significantly upregulated the short-chain fatty acid (SCFA)-producing bacterium *Flintibacter butyricus*. Conversely, the TTS intervention featured a distinct species-level regulatory landscape (Figure 8L). TTS-L selectively promoted *Bifidobacterium animalis* and exhibited the most robust restoration of *Paramuribaculum intestinale*, whereas TTS-H demonstrated the most potent inhibitory activity against *Ileibacterium valens* and *Faecalibaculum rodentium*. Notably, both DTS and TTS interventions resulted in elevated relative abundances of *Desulfovibrio porci* relative to the HFD baseline, suggesting a shared modulatory influence on this hydrogen sulfide-generating taxon, rather than a generalized inhibition. Collectively, these concentration-dependent, species-level compositional modulations indicate that DTS and TTS drive distinct, targeted remodeling of the gut microbiome, underscoring their divergent implications for host metabolic homeostasis.

LEfSe analysis (LDA > 2, *p* < 0.05) was employed to pinpoint the key discriminatory taxa underpinning the distinct clustering patterns observed via PLS-DA (Figure 8M,N, Tables S13 and S14). Consistent with the compositional alterations, HFD feeding significantly enriched the phylum Bacillota and its affiliated genus *Faecalibaculum*, alongside *Brotonthovivens* in the DTS-specific comparison, consolidating their status as obesity-associated pathobionts. The DTS intervention, notably at the DTS-H level, elicited a robust enrichment of multiple phyla, including Gemmatimonadota, Rhodothermota, Actinomycetota (class Acidimicrobiia), and Pseudomonadota (genera *Sphingomonas*, *Parasutterella*, and *Klebsiella*), in addition to the genus *Anaerotignum* within Bacillota. In contrast, DTSL selectively favored the expansion of Prevotellamassilia and Parabacteroides. Regarding the TTS intervention, TTS-L preferentially promoted the enrichment of Planctomycetota and Acidimicrobiaceae, whereas TTS-M significantly enhanced Acidobacteriota and Nocardioidaceae. Notably, TTS-H exhibited a unique discriminatory signature by preferentially enriching Cyanobacteriota (genus *Marmoreocelis*) and Flavobacteriia (genera *Salinirepens* and *Fulvivirga*). Conversely, the NC group was characterized by the persistent abundance of canonical beneficial commensals, including *Muribaculum*, *Duncaniella*, *Adlercreutzia*, and *Lactobacillus*. Collectively, these LEfSe outcomes corroborate the PLS-DA findings, illustrating that DTS and TTS orchestrate distinct, concentration-specific taxonomic selections rather than exerting a uniform restoration, thereby underscoring the intricate nuances of their respective microbiota-modulating potentials.

## 4. Discussion

Obesity, as a globally prevalent chronic metabolic disorder, serves as an independent risk factor for major non-communicable diseases, including type 2 diabetes, non-alcoholic fatty liver disease, and cardiovascular disease, and has become a critical public health concern worldwide [38]. Natural bioactive compounds have attracted substantial research interest in the prevention and treatment of obesity and its associated complications due to their favorable safety profiles and multi-target regulatory capabilities [39]. *Vigna angularis* (adzuki bean), a traditional medicinal and edible crop in China, has been documented to possess various biological activities including lipid regulation, hypoglycemic effects, and anti-inflammatory properties [24]. Previous studies have reported anti-obesity effects of adzuki bean saponin preparations and related red bean seedling extracts in HFD-fed mice [25,26,28,31]. However, these investigations treated saponins as bulk fractions, and the relative in vivo efficacy of saponin sub-fractions differing in glycosylation pattern has not been directly compared. In the present study, we therefore compared DTS and TTS side by side under identical dietary and dosing conditions, and characterized their differential effects on hepatic lipid metabolism and gut microbiota, providing fraction-level insight into how sugar-chain length may modulate the anti-obesity activity of *V. angularis* triterpenoid saponins. Both extracts significantly inhibited HFD-induced weight gain, ameliorated glucose and lipid metabolic disorders, and attenuated hepatic injury, yet exhibited distinct target preferences and pathway engagements, operating through a coordinated liver–gut axis.

LC-MS-based chemical characterization revealed that the major tentatively annotated saponins detected in DTS were disaccharide saponins (azukisaponins I and II, and angulasaponin A), whereas those detected in TTS were tri- and tetrasaccharide saponins (azukisaponins IV and VI, and yunganoside B1). Although the two extracts achieved statistically comparable anti-obesity efficacy at the matched dose levels, TTS tended to show numerically greater reductions in body weight gain, adiposity, and the associated metabolic indices, which did not reach statistical significance (Table S6); the statistically significant differences between the two extracts were confined to specific endpoints, including serum TG and ALT at the lower doses and several hepatic molecular markers (Table S6). Although the observed differences between DTS and TTS may be consistent with previous molecular simulation studies reporting that polysaccharide-chain saponins exhibit lower binding free energies with digestive enzymes [40,41], the heterogeneous nature of the extracts precludes definitive attribution of these effects to sugar-chain length alone.

Mechanistically, transcriptomic and Western blot analyses revealed that DTS and TTS treatment was associated with dual-directional changes in the PPARα/γ signaling axes, including upregulation of PPARα and its downstream oxidative targets, and downregulation of PPARγ and its lipogenic targets. HFD feeding severely suppressed hepatic fatty acid oxidation, evidenced by reduced PPARα and its downstream oxidative enzymes (ACSL1, ACOX1, Ehhadh). Both extracts effectively reversed this suppression and concurrently inhibited the PPARγ/FABP4 lipogenic axis. This “pro-degradation, anti-synthesis” regulation represents a classical anti-obesity mechanism of plant-derived triterpenoids [42,43]. Notably, recent studies have demonstrated that Panax notoginseng saponin (PNS) protects against diabetic cardiomyopathy by reducing lipotoxicity and oxidative stress while improving mitochondrial function, partly through upregulation of PPARα and PGC-1α [44]. Similarly, dietary red bean seedlings extract has been shown to alleviate obesity via activation of PPARα-AMPKα signaling [29]. These findings are consistent with our observation that both DTS and TTS upregulate hepatic PPARα and its downstream targets. Notably, transcriptomic KEGG analysis revealed intriguing pathway divergences: DTS-H preferentially enriched thermogenesis and carbon metabolism pathways, indicating robust pro-lipolytic and energy expenditure effects, whereas TTS-H significantly enriched the PPAR signaling pathway alongside peroxisomal fatty acid degradation and biosynthesis of unsaturated fatty acids. This divergence suggests that while both saponins are consistent with engagement of the core PPARα-associated β-oxidation machinery, DTS exerts more potent systemic thermogenic stimulation, whereas TTS engages peroxisomal β-oxidation as an auxiliary catabolic mechanism to achieve comprehensive lipid clearance.

Beyond hepatic regulation, 16S rRNA sequencing uncovered that DTS and TTS counteracted HFD-induced gut dysbiosis through distinct, concentration-specific microbial reconfiguration. HFD feeding depleted beneficial commensals, including *Muribaculum intestinale* and *Paramuribaculum intestinale*, and altered phylum-level community structure (an increase in the Firmicutes/Bacteroidetes ratio). At the genus level, DTS and TTS engaged clearly distinct compositional programs that more plausibly underlie their metabolic effects. DTS, across all intervention levels, suppressed the obesity-associated pro-inflammatory species *Ileibacterium valens* and *Faecalibaculum rodentium*, and selectively enriched *Turicimonas muris* together with the SCFA-producing species *Flintibacter butyricus*. Given that butyrate has been reported to serve as an endogenous ligand for PPARα and to activate fatty acid oxidation pathways [45,46], it is plausible that the restoration of SCFA producers by DTS contributed to the upregulated PPARα cascade observed in the liver, although direct SCFA quantification was not performed in this study. The response to TTS, in contrast, was non-linear and concentration-specific: TTS-L and TTS-M lowered the F/B ratio, with pronounced recovery of *Paramuribaculum intestinale*, whereas TTS-H sustained a high F/B ratio exceeding the HFD baseline and failed to reproduce the genus-level benefits observed at the lower doses. This concentration-specific adaptation underscores that DTS and TTS exert fundamentally different selective pressures on the gut ecosystem, potentially contributing to their divergent metabolic outcomes. Notably, the Firmicutes/Bacteroidetes (F/B) ratio, though historically linked to obesity, is now considered a controversial stand-alone marker of metabolic health [13,47]. We therefore report it as a descriptive phylum-level parameter only, and base our mechanistic interpretation primarily on genus-level biomarker taxa and inferred functional readouts such as SCFA producers.

LEfSe analysis further pinpointed the biomarker taxa driving these ecological shifts, corroborating the PLS-DA patterns. It is noteworthy that both DTS and TTS interventions resulted in elevated abundances of *Desulfovibrio porci* relative to the HFD baseline. While bacteria of the genus Desulfovibrio are generally associated with excessive hydrogen sulfide production and potential hepatic toxicity [48], the observed uniform elevation of this genus across both saponin treatments implies a shared structural shift in the microbiota rather than a generalized, targeted suppression of all pathogenic taxa. The profound inhibition of other obesity-associated pro-inflammatory species (*Ileibacterium valens* and *Faecalibaculum rodentium*) by both extracts, alongside the unique enrichment of *Bifidobacterium* (TTSL) and *Flintibacter* (DTS), illustrates that these interventions drive specific, tailored compositional remodeling. Consequently, we propose that the restored SCFA-producing microbiota and the hepatically activated PPARα pathway may engage in gut–liver crosstalk that contributes to the observed anti-obesity and hepatoprotective efficacy. However, because fecal and circulating SCFA levels, SCFA-related bacterial functional genes, and the direct causality of this link were not examined in the present study, this proposed mechanism remains hypothetical and warrants validation in future studies.

Several limitations of this study should be acknowledged. First, the experimental diet differed from the control diet in several components besides fat (Supplementary Table S3), and the observed phenotype therefore cannot be attributed specifically to the high-fat component. Dissecting the relative contribution of individual dietary components is beyond the scope of the present study. Second, while we observed significant alterations in gut microbiota composition and correlated them with metabolic improvements, the causal relationship between microbiota modulation and metabolic benefits requires confirmation through fecal microbiota transplantation (FMT) experiments. In addition, fecal and circulating SCFA levels were not directly measured, and the proposed SCFA–PPARα gut–liver axis therefore remains a hypothesis to be tested by targeted metabolomics and microbiota depletion or transplantation experiments. Third, although transcriptomics pointed to downstream PPARα/γ regulatory networks, specific inhibitors or gene knockout mice were not employed for definitive mechanistic validation to confirm whether DTS/TTS directly interacts with the ligand-binding domains of PPARα/γ. Future studies utilizing molecular docking, surface plasmon resonance, or fluorescence polarization competition binding assays are warranted to clarify the direct target engagement. Fourth, the potential synergistic effects of combined treatment with multiple extracts were not evaluated and warrant further exploration. Despite these limitations, our findings remain of interest. The multi-organ mechanisms by which DTS and TTS simultaneously target the liver, adipose tissue, and gut microbiota represent a mechanistic profile well suited to a systemic disease involving multiple interconnected organs and systems.

Given the rising global prevalence of obesity, the development of safe and effective natural anti-obesity bioactive compounds is of considerable importance. *Vigna angularis*, as a traditional medicinal and edible resource, offers advantages including high safety and abundant availability. However, as the present evidence derives exclusively from an HFD mouse model and in vitro adipocytes, the safety, bioavailability, and efficacy of these saponin extracts in humans remain to be established; our findings therefore constitute a preclinical foundation for future functional-food development rather than evidence of established anti-obesity interventions.

## 5. Conclusions

This study systematically evaluated the anti-obesity effects and differential molecular mechanisms of two triterpenoid saponin extracts, DTS and TTS, derived from *V. angularis* in HFD-induced obese mice. Our findings demonstrate that both extracts exert potent anti-obesity effects involving multi-target changes in hepatic lipid metabolism and gut microbiota remodeling. At the hepatic level, DTS and TTS were associated with upregulation of the PPARα-associated fatty acid β-oxidation cascade and downregulation of the PPARγ/FABP4 adipogenic axis, consistent with a ‘pro-catabolic, anti-synthetic’ dual regulatory mode. At the intestinal level, they restore gut microbial diversity, reshape phylum-level community structure, suppress obesity-associated pro-inflammatory pathobionts (*Faecalibaculum* and *Ileibacterium*), and enrich beneficial commensals (Bifidobacterium and SCFA-producing taxa such as *Flintibacter*), thereby counteracting HFD-induced gut dysbiosis. Notably, although the two extracts achieved comparable anti-obesity efficacy at the matched dose levels, TTS tended to show numerically greater improvements in body weight and adiposity-related indices, with statistically significant advantages confined to specific endpoints, a divergence that may be related to differences in their glycosylation patterns and selective effects on PPARα/γ signaling and specific microbial genera, although further studies with purified saponins are needed to establish a definitive structure–activity relationship. In summary, this study demonstrates that DTS and TTS attenuate HFD-induced obesity and related metabolic disorders in mice, providing a preclinical basis for their potential development as functional food ingredients, while acknowledging that human efficacy and safety remain to be established.

## Data Availability

The original contributions presented in this study are included in the article/Supplementary Materials. Further inquiries can be directed to the corresponding authors.

## Supplementary Materials

The following supporting information can be downloaded at: https://www.mdpi.com/article/10.3390/foods15183276/s1, Table S1. List of murine primers used for real-time polymerase chain reaction (cell); Table S2. Antibodies for Western blotting (cell); Table S3. Composition and energy content of experimental diets (g/kg diet); Table S4. List of murine primers used for real-time polymerase chain reaction (animal); Table S5. Antibodies for Western blotting (animal); Table S6. Direct statistical comparison between DTS and TTS at matched dose levels (Tukey’s HSD post hoc test); Table S7. Species relative abundance at the phylum level across sample groups (NC, HFD, DTSL, DTSM, DTSH, OT); Table S8. Species relative abundance at the phylum level across sample groups (NC, HFD, TTSL, TTSM, TTSH, OT); Table S9. Species relative abundance at the genus level across sample groups (NC, HFD, DTSL, DTSM, DTSH, OT); Table S10. Species relative abundance at the genus level across sample groups (NC, HFD, TTSL, TTSM, TTSH, OT); Table S11. Species-level relative abundance across sample groups (NC, HFD, DTSL, DTSM, DTSH, OT); Table S12. Species-level relative abundance across sample groups (NC, HFD, TTSL, TTSM, TTSH, OT); Table S13. LEfSe results: bacterial biomarkers significantly enriched in each group (NC, HFD, DTSL, DTSM, DTSH, OT); Table S14. EfSe results: bacterial biomarkers significantly enriched in each group (NC, HFD, TTSL, TTSM, TTSH, OT); Figure S1. Representative base peak ion chromatograms (BPI) of DTS (A) and TTS (B) extracts acquired in negative ion mode, and the mass spectra of the corresponding peaks 1–6 (C–H). The identified compounds and their MS data are listed as follows: 1. Angulasaponin A (RT: 18.122 min, *m*/*z* 977.1); 2. azukisaponin II (RT: 18.780 min, *m*/*z* 795.1); 3. azukisaponin I (RT: 19.061 min, *m*/*z* 779.2); 4. azukisaponin IV (RT: 15.174 min, *m*/*z* 971.3); 5. azukisaponin VI (RT: 16.191 min, m/z 1133.4); 6. yunganoside B1 (RT: 13.988 min, *m*/*z* 941.3); Figure S2. Hematoxylin and eosin (H&E) staining of (A) brown adipose tissue (BAT) and (B) epididymal white adipose tissue (WAT) from mice fed different diets (×40, magnification, 25 μm); Figure S3. Western blots of ACOX1, ACSL1, FABP4, PPARα, and PPARγ in differentiated 3T3-L1 adipocytes, with β-Actin or GADPH as a loading control; Figure S4. Western blots of AMPK, p-AMPK, FASN, and SREBP1c in HFD-fed mice, with β-Actin as a loading control.
