# Supplementary material for: Dual Modulation of Lipid Metabolism by Adzuki Bean (Vigna angularis) Saponins Involving PPARα/γ Signaling and Gut Microbiota Remodeling in Obesogenic Diet-Induced Obese Mice
Source: Foods. 2026 Sep 16;15(18):3276. doi: 10.3390/foods15183276 (PMC13606547; doi:10.3390/foods15183276)
Supplement: Supplementary file 1 [file foods-15-03276-s001.zip › foods-4540343-supplementary.pdf]

Dual Modulation of Lipid Metabolism by Adzuki Bean (*Vigna angularis*) Saponins Involving PPARα/γ Signaling and Gut Microbiota Remodeling in Obesogenic Diet-Induced Obese Mice

Qingfeng Guo [1], Jinhai Luo [2], Xia Zhang [1, 2], Hao Zhang [1, 3], Meiru Chen [1], Man Liu [1], Zhenhua Yin [1], Juanjuan Zhang [1], Baocheng Yang [1], Li Wang [1] Baojun Xu [2*], Lin Chen [1*]

[1] Henan Engineering Research Center of Chemistry and Biology of Medicinal Resources, Henan Comprehensive Utilization of Edible and Medicinal Plant Resources Engineering Technology Research Center, Zhengzhou Key Laboratory of Synthetic Biology of Natural Products, Henan Joint International Research Laboratory of Drug Discovery of Small Molecules, Huanghe Science and Technology College, Zhengzhou 450063, China;

[2] Food Science and Technology Program, Department of Life Sciences, Beijing Normal-Hong Kong Baptist University, Zhuhai 519087, China

[3] School of Pharmacy, Henan University, Kaifeng 475004, China

Corresponding Author:

E-mail: Lin Chen: lchenchina@163.com; Baojun Xu: baojunxu@bnbu.edu.cn

Table S1. List of murine primers used for real-time polymerase chain reaction（cell）

Symbol		Primer sequence（5’-3’）	Size（bp）
PPARG	F	ATTGAGTGCCGAGTCTGTGG	213
	R	GGCATTGTGAGACATCCCCA	
Cyp4a14	F	GCACCATCTGAAGGACAAGGAG	204
	R	AGCAAACCATAACCAATCCAGG	
Acsl1	F	TGGATTTCCTACAAAGAGGTGGCAG	171
	R	CGGGACGACCACCATTGAGTAAG	
Pparα	F	AAGTGCCTGTCTGTCGGGATG	85
	R	TTTCTGCTTTCAGTTTTGCTTTTTC	
Fabp4	F	ATGAAATCACCGCAGACGACAG	147
	R	TCATAACACATTCCACCACCAGC	
Plin5	F	AGAGCAAACACCGTACCCAG	217
	R	AGCTCCAGGGTCAAACTTCG	
Acox1	F	CCCCAAGACCCAAGAGTTCATT	166
	R	AATCTCACGGATAGGGACAACAAAG	
Ehhadh	F	GTTGGAGTTCCTGTTGCTCTTGAC	164
	R	CTGCGGGGTTCTATGGGTTTAC	
GAPDH	F	CGGTGCTGAGTATGTCGTGGAGTC	100
	R	GGCGGAGATGATGACCCTTTTG	

Table S2. Antibodies for western blotting（cell）

Target	Supplier	Catalog #	Host (raised in)
ACOX1	Proteintech	10957-1-AP	Rabbit
ACSL1	Proteintech	13989-1-AP	Rabbit
FABP4	Proteintech	12802-1-AP	Rabbit
PPARα	Proteintech	15540-1-AP	Rabbit
PPARγ	Proteintech	16643-1-AP	Rabbit
β-Actin	Proteintech	66009-1-Ig	Mouse
Anti-rabbit IgG (HRP)	Proteintech	RGAR001	Goat
Anti-mouse IgG (HRP)	Proteintech	RGAM001	Goat

Table S3. Composition and energy content of experimental diets (g/kg diet).

Ingredient (g/kg)	Control diet	High-fat diet
Corn	300	220
Wheat	200	100
Wheat bran	100	80
Soybean meal	180	100
Soybean oil	40	10
Fish meal	50	46.5

Sucrose	–	110
Egg yolk powder	–	190
Lard	–	120
Cholesterol	–	2
Sodium cholate	–	3
Cellulose (filler)	100	8.5
Mineral premix[1]	5	5
Vitamin premix[2]	5	5
**Total**	**1000**	**1000**
**Macronutrient composition**		
Protein (g/kg)	176.5	172.5
Fat (g/kg)	68.6	235.7
Digestible carbohydrate (g/kg)	454.0	410.0
Energy density (kcal/g)	3.14	4.45
Energy from protein (%)	22.5	15.5
Energy from fat (%)	19.7	47.8
Energy from carbohydrate (%)	57.8	36.7

[1] Mineral premix (per kg diet): Fe 80 mg, Cu 10 mg, Mn 60 mg, Zn 50 mg, Se 0.3 mg, I 0.5 mg, Co 0.2 mg.

[2] Vitamin premix (per kg diet): VA 8000 IU, VD_3 2000 IU, VE 50 IU, VK_3 2 mg, VB_1 2 mg, VB_2 6 mg, VB_6 3 mg, VB_{12} 0.03 mg, niacin 30 mg, pantothenic acid 12 mg, folic acid 1 mg, biotin 0.2 mg, choline chloride 800 mg.

Table S4. List of murine primers used for real-time polymerase chain reaction（animal）

Primer information	Symbol	Primers sequence (5'-3')	Size (bp)
NM_008084.4	M-GAPDH-S	CCTCGTCCCGTAGACAAAATG	133
	M-GAPDH-A	TGAGGTCAATGAAGGGGTCGT	
NM_001127330.2	M-pparg(2)-S	CAGAAGTGCCTTGCTGTGGG	187
	M-pparg(2)-A	TGGTCAGCGGGAAGGACTTTAT	
NM_024406.3	M-Fabp4(3)-S	AGCTCCTCCTCGAAGGTTTACAA	225
	M-Fabp4(3)-A	CAGTTTGAAGGAAATCTCGGTG	
NM_007822.2	M-cyp4A14(1)-S	CATGGCGGACTCTGTCAATA	189
	M-cyp4A14(1)-A	AGATCCTCGACAGCCTTAGT	
NM_001302163.2	M-ACSL1-S	TGCCACAGTGCTGACGTTTC	195
	M-ACSL1-A	CCCTTGGATGCCAGGTAATT	
NM_001077348.1	M-PLIN5-S	GCCATCTCGCCTATGAACACT	142
	M-PLIN5-A	GGATGGAAAGTAGGGCTAGGG	
NM_001271898.1	M-ACOX1-S	AGGGAATTTGGCATCGCAG	118
	M-ACOX1-A	GATTCAGCAAGGTAGGGATAAACA	
NM_023737.3	M-Ehhadh-S	CGTCTCCTCGGTTGGTGTTCTT	96
	M-Ehhadh-A	GACTCTACAGCAACCACAGGGA	

Table S5. Antibodies for western blotting（animal）

Target	Supplier	Catalog #	Host (raised in)
ACTIN	Servicebio	GB15003	Rabbit
PPAR gamma	Servicebio	GB152205	Rabbit
ACSL1	Servicebio	GB114032	Rabbit
FABP	Servicebio	GB111153	Rabbit
ACOX1	Servicebio	GB113313	Rabbit
PPAR alpha	Servicebio	GB15163	Rabbit
Anti-rabbit IgG (HRP)	Servicebio	GB23303	Goat

Table S6. Direct statistical comparison between DTS and TTS at matched dose levels (Tukey's HSD post hoc test)

Endpoint	Unit	DTS (30 mg/kg/day)	TTS (30 mg/kg/day)	p-value	DTS (60 mg/kg/day)	TTS (60 mg/kg/day)	p-value	DTS (120 mg/kg/day)	TTS (120 mg/kg/day)	p-value
Body weight gain	g	8.68 ± 0.34	7.19 ± 0.35	0.097	6.39 ± 0.44	7.34 ± 0.32	0.628	5.94 ± 0.30	5.46 ± 0.45	0.990
Obesity index	–	801.38 ± 14.29	766.50 ± 10.72	0.421	767.00 ± 13.42	772.62 ± 14.56	1.000	774.38 ± 10.02	752.25 ± 10.58	0.896
Liver coefficient	%	3.66 ± 0.14	3.73 ± 0.11	1.000	3.71 ± 0.14	3.16 ± 0.12	0.079	3.27 ± 0.13	2.78 ± 0.17	0.172
Adipose coefficient	%	2.54 ± 0.21	2.35 ± 0.06	0.991	2.09 ± 0.11	1.95 ± 0.14	0.999	1.81 ± 0.07	1.30 ± 0.14	0.286

Fasting blood glucose	mmol/L	7.07 ± 0.37	7.13 ± 0.10	1.000	7.10 ± 0.23	6.25 ± 0.18	0.227	6.23 ± 0.13	5.83 ± 0.08	0.950
AUC of OGTT	–	19.70 ± 0.25	21.42 ± 0.33	0.024	18.47 ± 0.29	19.69 ± 0.13	0.259	17.37 ± 0.26	16.36 ± 0.35	0.501
Triglycerides (TG)	mmol/L	1.44 ± 0.04	0.88 ± 0.04	<0.001	1.20 ± 0.02	0.65 ± 0.02	<0.001	0.45 ± 0.03	0.34 ± 0.05	0.679
Total cholesterol (TC)	mmol/L	6.37 ± 0.37	5.78 ± 0.55	0.915	4.38 ± 0.14	4.42 ± 0.17	1.000	2.88 ± 0.16	3.08 ± 0.19	1.000
LDL-cholesterol (LDL-C)	mmol/L	4.05 ± 0.25	3.83 ± 0.20	0.994	2.10 ± 0.22	1.40 ± 0.13	0.130	0.73 ± 0.11	0.72 ± 0.07	1.000
HDL-cholesterol (HDL-C)	mmol/L	1.00 ± 0.08	1.00 ± 0.08	1.000	1.54 ± 0.05	1.59 ± 0.10	1.000	2.05 ± 0.06	1.88 ± 0.06	0.821
Alanine aminotransferase (ALT)	U/L	27.42 ± 1.49	18.63 ± 1.06	<0.001	18.55 ± 1.17	12.38 ± 0.94	0.002	10.82 ± 0.42	9.36 ± 0.80	0.983
Aspartate aminotransferase (AST)	U/L	13.21 ± 0.71	13.09 ± 0.98	1.000	11.52 ± 0.81	14.51 ± 1.28	0.475	8.63 ± 0.98	6.61 ± 0.66	0.884

Additional direct comparison: TTS-H vs. positive control OT (body weight gain)

Endpoint	Unit	TTS-H (120 mg/kg/day)	OT (30 mg/kg/day)	p-value
Body weight gain	g	5.46 ± 0.45	7.10 ± 0.35	0.046

Data are expressed as mean ± SEM (n = 8 per group). All comparisons were performed within the same one-way ANOVA model including all nine groups (NC, Model, DTS-L/M/H, TTS-L/M/H, and OT), followed by Tukey's HSD post hoc test. ns, not significant ($p \geq 0.05$). The additional comparison between the TTS-H group and the positive control OT group is based on the same ANOVA model.

Table S7. Species relative abundance at the phylum level across sample groups (NC, HFD, DTSL, DTSM, DTSH, OT)

	DTSL	DTSM	DTSH	OT	HFD	NC
Bacillota	0.557589671862635	0.582886205621635	0.564562301530878	0.599759477984919	0.666059096519596	0.46816102383788
Fusobacteriota	5.42908147302943E-4	4.59902789175151E-4	8.19944073178824E-4	1.3717985650987E-4	3.848927387994E-4	3.07189171817993E-4
Bacteroidota	0.294651894657416	0.27105858800826	0.298369590975876	0.275278818058356	0.240217309555515	0.395725817123197
Chloroflexota	1.28825662071885E-4	1.93880587593446E-4	3.50727522631404E-4	1.64615827811844E-4	0.0	0.0
Campylobacterota	0.00580635662623995	0.0342086515830575	0.0103085454286933	0.00272987914454641	0.00784384857346364	0.0103971719692244
Acidobacteriota	0.00122844470618547	0.00134363756053132	9.5265178444476E-4	2.42351079834104E-4	4.42405446895862E-6	9.4519745174767E-6
Planctomycetota	1.24224745569317E-4	1.84862885844914E-4	1.70624200199062E-4	4.57266188366234E-6	0.0	0.0
Parcubacteria	0.0	0.0	0.0	0.0	0.0	0.0
Chlorobiota	9.20183300513462E-6	0.0	9.4791222332812E-6	4.57266188366234E-6	0.0	0.0
Spirochaetota	0.0	0.0	2.3697805583203E-5	0.0	8.84810893791724E-6	0.0
Verrucomicrobiota	0.0142260338259381	0.00265571316494278	0.00355467083748045	0.0468194850268187	0.0253055915624433	0.00517495604831849
Nitrospirota	4.60091650256731E-5	2.70531052455971E-5	1.42186833499218E-5	1.3717985650987E-5	0.0	0.0
Pseudomonadota	0.115050518063198	0.0870027864698403	0.0955116356225413	0.0558870735421211	0.0436388732818078	0.0959422673396473
Others	0.00178975651949868	6.31239122397266E-4	0.00780131759799043	0.00764549066948343	6.1051951671629E-4	0.0060634416529613
Deferribacterota	0.00100760071406224	0.00523026701414877	0.0049386226835395	6.8589928254935E-4	0.00126527957812217	0.00777897502788332
Gemmatimonadota	3.68073320205385E-5	3.60708069941295E-5	2.22759372482108E-4	7.31625901385974E-5	0.0	0.0
Deinococcota	4.60091650256731E-6	0.0	4.7395611166406E-6	4.57266188366234E-6	0.0	4.72598725873835E-6
Thermomicrobiota	4.60091650256731E-6	0.0	2.84373666998436E-5	9.14532376732467E-6	0.0	0.0
Actinomycetota	0.00604560428437345	0.0125255877287115	0.0105692212901085	0.00833596261391644	0.0136128156009857	0.00907389553677763

Latescibacteria	0.0	0.0	4.7395611166406E-6	4.57266188366234E-6	0.0	0.0
Cyanobacteriota	2.20843992123231E-4	1.48792078850784E-4	2.22759372482108E-4	5.6701007357413E-4	2.21202723447931E-5	1.89039490349534E-5
Rhodothermota	0.0	0.0	4.7395611166406E-5	0.0	0.0	0.0
BRC1	0.0	0.0	0.0	0.0	0.0	0.0
Synergistota	0.0	2.70531052455971E-5	0.0	1.3717985650987E-5	0.0	1.89039490349534E-5
Mycoplasmatota	5.24504481292674E-4	3.69725771689827E-4	4.69216550547419E-4	1.23461870858883E-4	1.23873525130841E-4	1.98491464867011E-4
Elusimicrobiota	0.0	0.0	2.3697805583203E-5	0.0	0.0	0.0
CandidatusSaccharibacteria	9.61591549036568E-4	0.00100998259583563	0.00101900564007773	0.00149526043595758	9.02507111667559E-4	0.00112478496757973

Table S8 Species relative abundance at the phylum level across sample groups (NC, HFD, TTSL, TTSM, TTSH, OT)

	TTSL	TTSM	TTSH	OT	HFD	NC
Thermomicrobiota	3.67522074294587E-5	5.12111435448354E-5	3.17515036604948E-5	9.14532376732467E-6	0.0	0.0
BRC1	0.0	0.0	4.5359290943564E-6	0.0	0.0	0.0
Gemmatimonadota	2.1591921864807E-4	1.67600106146734E-4	1.95044951057325E-4	7.31625901385974E-5	0.0	0.0
Synergistota	2.7564155572094E-5	2.79333510244556E-5	0.0	1.3717985650987E-5	0.0	1.89039490349534E-5
Pseudomonadota	0.0770280327462168	0.0824685633412012	0.0742486233455199	0.0558870735421211	0.0436388732818078	0.0959422673396473
Fusobacteriota	4.36432463224822E-4	3.67789121821999E-4	2.90299462038809E-4	1.3717985650987E-4	3.848927387994E-4	3.07189171817993E-4
Cyanobacteriota	3.85898178009317E-4	1.7225566465081E-4	3.71946185737225E-4	5.6701007357413E-4	2.21202723447931E-5	1.89039490349534E-5
Bacillota	0.59842241149609	0.603881804680699	0.669630140341646	0.599759477984919	0.666059096519596	0.46816102383788
Elusimicrobiota	0.0	1.39666755122278E-5	0.0	0.0	0.0	0.0
CandidatusSaccharibacteria	0.00102906180802484	0.00174583443902848	0.00112944634449474	0.00149526043595758	9.02507111667559E-4	0.00112478496757973
Latescibacteria	4.59402592868234E-6	4.65555850407594E-6	1.36077872830692E-5	4.57266188366234E-6	0.0	0.0
Deinococcota	1.3782077786047E-5	4.65555850407594E-6	9.07185818871279E-6	4.57266188366234E-6	0.0	4.72598725873835E-6
Bacteroidota	0.266072199711495	0.274766407352058	0.202565521495768	0.275278818058356	0.240217309555515	0.395725817123197
Rhodothermota	1.83761037147294E-5	2.32777925203797E-5	0.0	0.0	0.0	0.0

Campylobacterota	0.0247204535222397	0.0110197069791478	0.0186517404359935	0.00272987914454641	0.00784384857346364	0.0103971719692244
Acidobacteriota	8.13142589376774E-4	8.19378296717366E-4	5.57919278605837E-4	2.42351079834104E-4	4.42405446895862E-6	9.4519745174767E-6
Actinomycetota	0.0130516276633865	0.00691815993705685	0.0101423374549809	0.00833596261391644	0.0136128156009857	0.00907389553677763
Parcubacteria	0.0	0.0	0.0	0.0	0.0	0.0
Deferribacterota	0.00893997445721584	0.0115364739731002	0.016252233945079	6.8589928254935E-4	0.00126527957812217	0.00777897502788332
Verrucomicrobiota	0.00646838850758474	0.00227656810849314	0.00302092877684136	0.0468194850268187	0.0253055915624433	0.00517495604831849
Planctomycetota	1.47008829717835E-4	1.86222340163038E-5	4.08233618492076E-5	4.57266188366234E-6	0.0	0.0
Chlorobiota	0.0	0.0	4.5359290943564E-6	4.57266188366234E-6	0.0	0.0
Others	8.68270900520963E-4	0.0015875454498899	0.00142428173562791	0.00764549066948343	6.1051951671629E-4	0.0060634416529613
Spirochaetota	0.0	4.65555850407594E-6	0.0	0.0	8.84810893791724E-6	0.0
Chloroflexota	3.95086229866681E-4	3.72444680326075E-4	3.94625831209007E-4	1.64615827811844E-4	0.0	0.0
Mycoplasmatota	8.72864926449645E-4	0.00170393441249179	9.75224755286625E-4	1.23461870858883E-4	1.23873525130841E-4	1.98491464867011E-4
Nitrospirota	3.21581815007764E-5	4.65555850407594E-5	4.5359290943564E-5	1.3717985650987E-5	0.0	0.0

Table S9 Species relative abundance at the genus level across sample groups (NC, HFD, DTSL, DTSM, DTSH, OT)

	DTSL	DTSM	DTSH	OT	HFD	NC
Lacrimispora	0.0222500322064155	0.0178730848655912	0.0160244561353619	0.0167679511273898	0.0124625614390564	0.0450055766649653
Anaerotignum	0.00432946242891584	0.00413912510257636	0.011882079719418	0.00585300721108779	0.0030260532567677	0.00458893362823494
Roseburia	0.00369913686806412	0.00320128412072899	0.00512820512820513	0.00521740720925873	0.0129978720298004	0.00145087808843267
Others	0.563879124721644	0.576204088625973	0.61396748661074	0.59156984055128	0.562483133292337	0.521890772982476
Anaerostipes	0.00369453595156155	0.00683090907451327	0.00317076638703256	0.00575240864964722	0.00378256657095962	0.0104491578290705
Muribaculum	0.0300761911772825	0.0454447079617289	0.056614057538272	0.0508251368369069	0.0306719696332901	0.0930972230098868
Laedolimicola	0.0128181533761525	0.00486054124245895	0.0058723162235177	0.00491561152493701	0.0239120144047214	0.0022306659861245
Duncaniella	0.0580267589303789	0.0344836914863878	0.0435755249063937	0.0398553209780009	0.0314727234921716	0.0957059679767103
Paramuribaculum	0.0431519958775788	0.031489814505875	0.0440731788236409	0.0342675281561656	0.0203727708295545	0.0598688065936974

Turicimonas	0.0272650311942139	0.0493403551170949	0.0428219346888478	0.0206547137285028	0.021815012586435	0.0476190476190476
Acetatifactor	0.00332646263135617	0.00439612960240953	0.0164225792691597	0.00384103598227636	0.00644584736127271	0.00305298776914497
Allobaculum	0.0408883449583157	0.0397229762022851	0.0308403241859804	0.0440164432921337	0.0305215517813455	0.0146741904383826
Ruminococcus	0.00110882087711872	0.00508147493529799	0.0139485283662733	0.00933737556643849	0.00105292496361215	0.00479215108036069
Blautia	0.0245366877081915	0.00126247824479453	0.00227498933598749	0.00142209784581899	0.00108389334489486	0.00126656458534188
Desulfovibrio	0.0157397353552828	0.0140405616224649	0.020242665529172	0.0143215770196304	0.00902507111667559	0.0144520690372219
Faecalibaculum	0.0122568415628393	0.0205107626270369	0.00248826958623631	0.00416569497601639	0.0423957139760305	4.53694776838882E-4
Ileibacterium	0.042310028157609	0.0531728783602211	0.00747428788094223	0.0408247252973373	0.0823758942120095	0.0345280629123424
Helicobacter	0.00576494837771684	0.034199633881309	0.0102374520119437	0.00272530648266275	0.00783942451899468	0.0103357341348608
Akkermansia	0.0142214329094356	0.00263316891057145	0.00353097303189725	0.0468194850268187	0.0253055915624433	0.00517495604831849
Kineothrix	0.0523538288827134	0.0340508418024582	0.0290440305227736	0.0358679598154474	0.0604016156646921	0.0138754985916558
Flintibacter	0.0162274325045549	0.00821061744203872	0.013820560216124	0.0153595712672218	0.00474258639072364	0.0127648915858523
Bifidobacterium	0.00207501334265786	0.00885087426618452	0.00654533390208067	0.00561980145502101	0.00581320757221163	0.00272216866103329

Table S10 Species relative abundance at the genus level across sample groups (NC, HFD, TTSL, TTSM, TTSH, OT)

	TTSL	TTSM	TTSH	OT	HFD	NC
Acetatifactor	0.0127346398743075	0.0135057752203243	0.0120519636037049	0.00384103598227636	0.00644584736127271	0.00305298776914497
Ileibacterium	0.0455681431866001	0.0208382798642439	0.0138164400214096	0.0408247252973373	0.0823758942120095	0.0345280629123424
Paramuribaculum	0.0664985253176769	0.0472166743483382	0.0264807540528526	0.0342675281561656	0.0203727708295545	0.0598688065936974
Akkermansia	0.00645001240387001	0.00227656810849314	0.00301185691865265	0.0468194850268187	0.0253055915624433	0.00517495604831849
Helicobacter	0.0247112654703823	0.0109778069526111	0.0186109170741443	0.00272530648266275	0.00783942451899468	0.0103357341348608
Lawsonibacter	0.00758473680825455	0.00531664781165473	0.00405058468126026	0.00576155397341454	0.001924463693997	0.00664946407304486
Muribaculum	0.0408133263504139	0.0337295213620302	0.0395623735609765	0.0508251368369069	0.0306719696332901	0.0930972230098868
Flintibacter	0.0148662679052161	0.01569388771724	0.0124012301439704	0.0153595712672218	0.00474258639072364	0.0127648915858523
Turicimonas	0.0358288082177936	0.0313365642909352	0.0141702424907694	0.0206547137285028	0.021815012586435	0.0476190476190476

Sporofaciens	0.0034087672390823	0.00453451398296997	0.00529342925311391	0.00873835685967872	0.00407013011144193	0.00480160305487816
Bifidobacterium	0.00981283938366548	0.0033287243304143	0.00792426812784063	0.00561980145502101	0.00581320757221163	0.00272216866103329
Mucispirillum	0.00884809393864219	0.011466640595539	0.0161388357177201	6.72181296898363E-4	0.00126527957812217	0.00776007107884837
Faecalibaculum	0.0109521578139787	0.00223001252345238	0.00193230579419583	0.00416569497601639	0.0423957139760305	4.53694776838882E-4
Laedolimicola	0.00816358407526852	0.00951130602382715	0.00697172301802578	0.00491561152493701	0.0239120144047214	0.0022306659861245
Anaerotignum	0.00647298253351342	0.0079144494569291	0.0073028458419138	0.00585300721108779	0.0030260532567677	0.00458893362823494
Allobaculum	0.0174527045030642	0.0217787026820673	0.00728016619644202	0.0440164432921337	0.0305215517813455	0.0146741904383826
Others	0.536058509514227	0.600040968914836	0.670405784216781	0.587157221833546	0.557360078217283	0.508738350441407
Desulfovibrio	0.0170851824287696	0.0236921372272425	0.0170460215365913	0.0143215770196304	0.00902507111667559	0.0144520690372219
Anaerostipes	0.00255427841634738	0.0121835966051667	0.00897660367773131	0.00575240864964722	0.00378256657095962	0.0104491578290705
Roseburia	0.00407949502466992	0.005456314566777	0.00396893795756185	0.00521740720925873	0.0129978720298004	0.00145087808843267
Duncaniella	0.0227312402951202	0.0248048157097166	0.0176991953261787	0.0398553209780009	0.0314727234921716	0.0957059679767103
Kineothrix	0.0469693210948483	0.0295441742668659	0.0343006958115231	0.0358679598154474	0.0604016156646921	0.0138754985916558
Lacrimispora	0.0503551182042871	0.0626219174383255	0.05060282497664	0.0167679511273898	0.0124625614390564	0.0450055766649653

Table S11 species-level relative abundance across sample groups (NC, HFD, DTSL, DTSM, DTSH, OT).

	DTSL	DTSM	DTSH	OT	HFD	NC
Others	0.687786407052284	0.658174997520131	0.706502677852031	0.663058836440457	0.628596203276454	0.67972929544982
Akkermansia_muciniphila	*0.0142214329094356*	*0.00263316891057145*	*0.00353097303189725*	*0.0468194850268187*	*0.0253055915624433*	*0.00517495604831849*
Muribaculum_intestinale	*0.00917422750611922*	*0.0111954767208029*	*0.0296791317124034*	*0.0398233123448153*	*0.0117414405606162*	*0.0743492315544717*
Turicimonas_muris	*0.0272650311942139*	*0.0493403551170949*	*0.0428219346888478*	*0.0206547137285028*	*0.021815012586435*	*0.0476190476190476*
Muribaculum_gordoncarteri	*0.0123442589763881*	*0.0148070662710901*	*0.021389639319399*	*0.007393994265882*	*0.0107548764140384*	*0.0064084387228492*
Allobaculum_stercoricanis	*0.0408883449583157*	*0.0397229762022851*	*0.0308403241859804*	*0.0440164432921337*	*0.0305215517813455*	*0.0146741904383826*
Kineothrix_alysoides	*0.0523538288827134*	*0.0340508418024582*	*0.0290440305227736*	*0.0358679598154474*	*0.0604016156646921*	*0.0138754985916558*
Acetatifactor_muris	*0.00332646263135617*	*0.00439612960240953*	*0.0164225792691597*	*0.00384103598227636*	*0.00644584736127271*	*0.00305298776914497*

Laedolimicola_ammoniilytica	0.0128181533761525	0.00486054124245895	0.0058723162235177	0.00491561152493701	0.0239120144047214	0.0022306659861245
Faecalibaculum_rodentium	0.0122568415628393	0.0205107626270369	0.00248826958623631	0.00416569497601639	0.0423957139760305	4.53694776838882E-4
Flintibacter_butyricus	0.0102416401347148	0.00506794838267519	0.00862126167116925	0.00879322880228267	0.00289333162269894	0.00827047770279211
Helicobacter_ganmani	0.00538307230800375	0.033284337153833	0.00960235082231385	0.00248752806471231	0.00673783495622398	0.00913060738388249
Duncaniella_dubosii	0.00529105397795241	0.00479290847934495	0.00705720650267785	0.00723852376183748	0.00506996642142658	0.0063706308247793
Ileibacterium_valens	0.042310028157609	0.0531728783602211	0.00747428788094223	0.0408247252973373	0.0823758942120095	0.0345280629123424
Duncaniella_muris	0.011175626184736	0.0220572984769102	0.0190672543722451	0.0226163856765939	0.0131128974459934	0.0239796593508384
Desulfovibrio_porci	0.0100115943095865	0.0104424986248005	0.0155125835347647	0.0132149928437842	0.00754743692404341	0.0102837482750147
Paramuribaculum_intestinale	0.0431519958775788	0.031489814505875	0.0440731788236409	0.0342675281561656	0.0203727708295545	0.0598688065936974

Table S12 species-level relative abundance across sample groups (NC, HFD, TTSL, TTSM, TTSH, OT).

	TTSL	TTSM	TTSH	OT	HFD	NC
Helicobacter_ganmani	0.016400672565396	0.00771426044125384	0.0172637461331204	0.00248752806471231	0.00673783495622398	0.00913060738388249
Ileibacterium_valens	0.0455681431866001	0.0208382798642439	0.0138164400214096	0.0408247252973373	0.0823758942120095	0.0345280629123424
Laedolimicola_ammoniilytica	0.00816358407526852	0.00951130602382715	0.00697172301802578	0.00491561152493701	0.0239120144047214	0.0022306659861245
Turicimonas_muris	0.0358288082177936	0.0313365642909352	0.0141702424907694	0.0206547137285028	0.021815012586435	0.0476190476190476
Muribaculum_intestinale	0.0176318715142828	0.0105075955436994	0.0298917727318087	0.0398233123448153	0.0117414405606162	0.0743492315544717
Bifidobacterium_animalis	0.00953719782794454	0.00256986829424992	0.00758860937485825	0.00533629641823395	0.00560527701217057	0.00235826764211044
Kineothrix_alysoides	0.0469693210948483	0.0295441742668659	0.0343006958115231	0.0358679598154474	0.0604016156646921	0.0138754985916558
Mucispirillum_schaedleri	0.00884809393864219	0.011466640595539	0.0161388357177201	6.72181296898363E-4	0.00126527957812217	0.00776007107884837
Akkermansia_muciniphila	0.00645001240387001	0.00227656810849314	0.00301185691865265	0.0468194850268187	0.0253055915624433	0.00517495604831849
Allobaculum_stercoricanis	0.0174527045030642	0.0217787026820673	0.00728016619644202	0.0440164432921337	0.0305215517813455	0.0146741904383826
Acetatifactor_muris	0.0127346398743075	0.0135057752203243	0.0120519636037049	0.00384103598227636	0.00644584736127271	0.00305298776914497
Duncaniella_muris	0.0121603866332222	0.0149908983831245	0.00803766635519954	0.0226163856765939	0.0131128974459934	0.0239796593508384
Muribaculum_gordoncarteri	0.0103916866506795	0.0144368869211395	0.00777458246772686	0.007393994265882	0.0107548764140384	0.0064084387228492

Others	0.648345691263081	0.722472846455025	0.766327076775135	0.655550525627484	0.622725482996146	0.671179984498762
Faecalibaculum_rodentium	0.0109521578139787	0.00223001252345238	0.00193230579419583	0.00416569497601639	0.0423957139760305	4.53694776838882E-4
Sporofaciens_musculi	0.0034087672390823	0.00453451398296997	0.00529342925311391	0.00873835685967872	0.00407013011144193	0.00480160305487816
Flintibacter_butyricus	0.0098082453577368	0.0113362849574249	0.00840961254093676	0.00879322880228267	0.00289333162269894	0.00827047770279211
Desulfovibrio_porci	0.0128494905225245	0.0217321470970265	0.0132585207428037	0.0132149928437842	0.00754743692404341	0.0102837482750147
Paramuribaculum_intestinale	0.0664985253176769	0.0472166743483382	0.0264807540528526	0.0342675281561656	0.0203727708295545	0.0598688065936974

Table S13 LEfSe results: bacterial biomarkers significantly enriched in each group (NC, HFD, DTSL, DTSM, DTSH, OT).

Species	Group	LDA value	P value
k__Bacteria.p__Gemmatimonadota.c__Gemmatimonadia.o__Gemmatimonadales.f__Gemmatimonadaceae	DTSH	3.09056812359	0.00335639038277
k__Bacteria.p__Pseudomonadota.c__Betaproteobacteria.o__Burkholderiales.f__Sutterellaceae.g__Parasutterella	DTSH	3.70665151813	0.00456882880434
k__Bacteria.p__Pseudomonadota.c__Alphaproteobacteria.o__Caulobacterales.f__Caulobacteraceae.g__Phenylobacterium	DTSH	3.45502372069	0.0030907389766
k__Bacteria.p__Pseudomonadota.c__Gammaproteobacteria.o__Enterobacterales.f__Enterobacteriaceae.g__Klebsiella	DTSH	3.0535638314	0.0352096867369
k__Bacteria.p__Actinomycetota.c__Acidimicrobiia.o__Acidimicrobiales.f__Iamiaceae	DTSH	3.2859887587	1.55551679157E-4
k__Bacteria.p__Bacteroidota.c__Bacteroidia	NC	5.12357402559	0.00348611694084
k__Bacteria.p__Pseudomonadota.c__Gammaproteobacteria	NC	3.64213868662	0.00821929241284
k__Bacteria.p__Bacteroidota.c__Bacteroidia.o__Bacteroidales.f__Prevotellaceae.g__Prevotellamassilia	DTSL	3.17451137853	0.00938182612166
k__Bacteria.p__Rhodothermota	DTSH	3.50303257757	4.86970142659E-4
k__Bacteria.p__Pseudomonadota.c__Gammaproteobacteria.o__Enterobacterales	NC	3.64964189389	0.00131328047398
k__Bacteria.p__Pseudomonadota.c__Alphaproteobacteria.o__Caulobacterales	DTSH	3.38576948684	0.0258453551014
k__Bacteria.p__Actinomycetota.c__Acidimicrobiia.o__Acidimicrobiales.f__Iamiaceae.g__Actinomarinicola	DTSH	3.57880460677	0.00667973585357
k__Bacteria.p__Gemmatimonadota.c__Gemmatimonadia.o__Gemmatimonadales	DTSH	3.05792287681	0.00335639038277
k__Bacteria.p__Pseudomonadota.c__Alphaproteobacteria.o__Sphingomonadales.f__Sphingomonadaceae.g__Sphingomonas	DTSH	3.34609742831	8.22823334243E-4
k__Bacteria.p__Bacteroidota.c__Cytophagia.o__Cytophagales.f__Fulvivirgaceae.g__Ohtaekwangia	DTSH	3.56940960466	0.00338188710367

k__Bacteria.p__Actinomycetota.c__Actinobacteria.o__Micrococcales	DTSL	2.82533727146	0.0051218102846
k__Bacteria.p__Gemmatimonadota.c__Longimicrobia.o__Longimicrobiales.f__Longimicrobiaceae	DTSH	3.21090127458	7.80595265488E-4
k__Bacteria.p__Gemmatimonadota	DTSH	3.21547966878	8.34237336396E-4
k__Bacteria.p__Actinomycetota.c__Acidimicrobiia	DTSH	3.21818444929	1.71972372021E-4
k__Bacteria.p__Gemmatimonadota.c__Gemmatimonadia.o__Gemmatimonadales.f__Gemmatimonadaceae.g__Roseisolibacter	DTSH	3.20106469129	0.00335639038277
k__Bacteria.p__Actinomycetota.c__Actinobacteria.o__Streptomycetales.f__Streptomycetaceae	DTSH	3.02463409567	1.996513371E-4
k__Bacteria.p__Bacteroidota.c__Bacteroidia.o__Bacteroidales.f__Muribaculaceae	NC	5.09847775717	0.0101548724097
k__Bacteria.p__Rhodothermota.c__Rhodothermia.o__Rhodothermales.f__Salisaetaceae.g__Roseithermus	DTSH	3.50408643313	4.86970142659E-4
k__Bacteria.p__Gemmatimonadota.c__Longimicrobia.o__Longimicrobiales	DTSH	3.21062338052	7.80595265488E-4
k__Bacteria.p__Bacillota.c__Clostridia.o__Eubacteriales.f__Lachnospiraceae.g__Anaerotignum	DTSH	3.91215806709	0.0150536532306
k__Bacteria.p__Bacteroidota	NC	5.12357402594	0.00348611694084
k__Bacteria.p__Actinomycetota.c__Actinobacteria.o__Streptomycetales	DTSH	3.04862776592	1.996513371E-4
k__Bacteria.p__Pseudomonadota.c__Alphaproteobacteria.o__Hyphomicrobiales	DTSM	3.19024485671	1.89981909206E-4
k__Bacteria.p__Pseudomonadota.c__Gammaproteobacteria.o__Enterobacterales.f__Enterobacteriaceae	NC	3.65385000003	0.00133095476518
k__Bacteria.p__Actinomycetota.c__Actinobacteria.o__Streptomycetales.f__Streptomycetaceae.g__Streptomyces	DTSH	3.02905988006	1.996513371E-4
k__Bacteria.p__Actinomycetota.c__Acidimicrobiia.o__Acidimicrobiales.f__Iamiaceae.g__Rhabdothermincola	DTSH	3.37563044467	0.00195266482332
k__Bacteria.p__Bacteroidota.c__Bacteroidia.o__Bacteroidales.f__Porphyromonadaceae.g__Parabacteroides	DTSL	3.35217846648	0.00387164124986
k__Bacteria.p__Rhodothermota.c__Rhodothermia.o__Rhodothermales.f__Salisaetaceae	DTSH	3.50174763357	4.86970142659E-4
k__Bacteria.p__Pseudomonadota.c__Alphaproteobacteria.o__Hyphomicrobiales.f__Hyphomicrobiaceae	DTSL	3.6226444028	0.0100545795542
k__Bacteria.p__Bacteroidota.c__Bacteroidia.o__Bacteroidales.f__Porphyromonadaceae	DTSL	3.35156614942	0.00387164124986
k__Bacteria.p__Gemmatimonadota.c__Longimicrobia.o__Longimicrobiales.f__Longimicrobiaceae.g__Longimicrobium	DTSH	3.21347559003	7.80595265488E-4
k__Bacteria.p__Actinomycetota.c__Thermoleophilia	DTSL	3.20307948389	1.33945958826E-4
k__Bacteria.p__Rhodothermota.c__Rhodothermia.o__Rhodothermales	DTSH	3.50848362035	4.86970142659E-4
k__Bacteria.p__Gemmatimonadota.c__Longimicrobia	DTSH	3.21216143972	7.80595265488E-4
k__Bacteria.p__Rhodothermota.c__Rhodothermia	DTSH	3.50438320113	4.86970142659E-4

k__Bacteria.p__Gemmatimonadota.c__Gemmatimonadia	DTSH	3.06547638835	0.00335639038277
k__Bacteria.p__Pseudomonadota.c__Alphaproteobacteria.o__Hyphomicrobiales.f__Hyphomicrobiaceae.g__Pedomicrobium	DTSL	3.68057750694	0.0078289492453
k__Bacteria.p__Bacillota.c__Clostridia.o__Eubacteriales.f__Lachnospiraceae.g__Brotonthovivens	HFD	3.38212394078	0.0460794137856
k__Bacteria.p__Actinomycetota.c__Acidimicrobiia.o__Acidimicrobiales	DTSH	3.21226918357	1.71972372021E-4
k__Bacteria.p__Bacteroidota.c__Bacteroidia.o__Bacteroidales	NC	5.12357402559	0.00348611694084
k__Bacteria.p__Pseudomonadota.c__Alphaproteobacteria	DTSM	3.31144148997	1.34928063641E-4
k__Bacteria.p__Actinomycetota.c__Actinobacteria.o__Streptosporangiales	DTSH	2.75403850754	7.60196197489E-5

Table S14 EfSe results: bacterial biomarkers significantly enriched in each group (NC, HFD, TTSL, TTSM, TTSH, OT).

Species	Group	LDA value	P value
k__Bacteria.p__Actinomycetota.c__Actinobacteria.o__Propionibacteriales.f__Nocardioidaceae	TTSM	3.37417849156	0.00200121103986
k__Bacteria.p__Bacteroidota.c__Bacteroidia	NC	5.12648073338	0.00243188778048
k__Bacteria.p__Acidobacteriota	TTSM	3.07487646543	3.41864560957E-4
k__Bacteria.p__Bacteroidota.c__Bacteroidia.o__Bacteroidales.f__Muribaculaceae.g__Muribaculum	NC	4.66902107668	0.00543503967161
k__Bacteria.p__Pseudomonadota.c__Gammaproteobacteria	NC	3.67476438406	0.00339576434589
k__Bacteria.p__Bacteroidota.c__Bacteroidia.o__Bacteroidales.f__Prevotellaceae.g__Prevotellamassilia	TTSM	2.87891689068	0.0203871760874
k__Bacteria.p__Pseudomonadota.c__Gammaproteobacteria.o__Xanthomonadales.f__Rhodanobacteraceae	TTSL	2.88438081928	0.00667973585357
k__Bacteria.p__Pseudomonadota.c__Gammaproteobacteria.o__Enterobacterales	NC	3.67902706449	3.84633609421E-4
k__Bacteria.p__Cyanobacteriota.c__Cyanophyceae.o__Coleofasciculales.f__Coleofasciculaceae	TTSH	3.37573510473	4.86970142659E-4
k__Bacteria.p__Actinomycetota.c__Acidimicrobiia.o__Acidimicrobiales.f__Acidimicrobiaceae	TTSL	3.03622907142	0.00667973585357
k__Bacteria.p__Pseudomonadota.c__Alphaproteobacteria.o__Sphingomonadales.f__Sphingomonadaceae.g__Sphingomonas	TTSM	2.85670039879	2.92809683447E-4
k__Bacteria.p__Bacteroidota.c__Cytophagia.o__Cytophagales.f__Fulvivirgaceae.g__Ohtaekwangia	TTSM	3.51484440136	0.0021954224592
k__Bacteria.p__Planctomycetota.c__Planctomycetia.o__Isosphaerales.f__Isosphaeraceae.g__Aquisphaera	TTSL	2.94103663383	0.00667973585357

k__Bacteria.p__Actinomycetota.c__Actinobacteria.o__Propionibacteriales.f__Nocardioidaceae.g__Nocardioides	TTSM	2.90658471658	0.00260391164605
k__Bacteria.p__Pseudomonadota.c__Gammaproteobacteria.o__Xanthomonadales.f__Rhodanobacteraceae.g__Rhodanobacter	TTSL	2.92208568561	0.00667973585357
k__Bacteria.p__Bacillota.c__Erysipelotrichia.o__Erysipelotrichales.f__Erysipelotrichaceae.g__Faecalibaculum	HFD	4.55693713641	0.0020608545488
k__Bacteria.p__Bacteroidota.c__Flavobacteriia.o__Flavobacteriales.f__Flavobacteriaceae	TTSH	3.06308746005	0.0013576499906
k__Bacteria.p__Planctomycetota.c__Planctomycetia	TTSL	2.59427359606	0.00667973585357
k__Bacteria.p__Pseudomonadota.c__Alphaproteobacteria.o__Hyphomicrobiales.f__Roseiarcaceae	TTSL	2.78482550381	0.00667973585357
k__Bacteria.p__Actinomycetota.c__Acidimicrobiia	TTSM	3.11843159748	2.36060995306E-4
k__Bacteria.p__Bacteroidota.c__Bacteroidia.o__Bacteroidales.f__Muribaculaceae	NC	5.10122686985	0.00996204718663
k__Bacteria.p__Actinomycetota.c__Coriobacteriia.o__Eggerthellales	NC	3.3275430972	0.00566322169821
k__Bacteria.p__Bacteroidota.c__Flavobacteriia.o__Flavobacteriales.f__Crocinitomicaceae.g__Salinirepens	TTSH	3.23111439408	4.86970142659E-4
k__Bacteria.p__Bacteroidota	NC	5.12648072765	0.00243188778048
k__Bacteria.p__Actinomycetota.c__Acidimicrobiia.o__Acidimicrobiales.f__Acidimicrobiaceae.g__Acidiferrimicrobium	TTSL	3.09358849653	0.00667973585357
k__Bacteria.p__Pseudomonadota.c__Gammaproteobacteria.o__Enterobacterales.f__Enterobacteriaceae	NC	3.68151781549	3.29607496672E-4
k__Bacteria.p__Bacillota	HFD	5.15931707165	0.0376715500442
k__Bacteria.p__Bacteroidota.c__Bacteroidia.o__Bacteroidales.f__Porphyromonadaceae.g__Parabacteroides	TTSM	3.28171737153	0.00587528566149
k__Bacteria.p__Actinomycetota.c__Coriobacteriia.o__Eggerthellales.f__Eggerthellaceae	NC	3.3275430972	0.00566322169821
k__Bacteria.p__Actinomycetota.c__Coriobacteriia.o__Eggerthellales.f__Eggerthellaceae.g__Adlercreutzia	NC	3.32748652662	0.00566322169821
k__Bacteria.p__Bacteroidota.c__Bacteroidia.o__Bacteroidales.f__Muribaculaceae.g__Duncaniella	NC	4.79194168795	0.00311766502187
k__Bacteria.p__Bacteroidota.c__Bacteroidia.o__Bacteroidales.f__Porphyromonadaceae	TTSM	3.281107125	0.00587528566149
k__Bacteria.p__Pseudomonadota.c__Alphaproteobacteria.o__Hyphomicrobiales.f__Roseiarcaceae.g__Roseiarcus	TTSL	2.65211267699	0.00667973585357
k__Bacteria.p__Pseudomonadota.c__Betaproteobacteria.o__Burkholderiales.f__Burkholderiaceae.g__Paraburkholderia	TTSL	3.27453497891	0.00667973585357
k__Bacteria.p__Pseudomonadota.c__Gammaproteobacteria.o__Xanthomonadales.f__Xanthomonadaceae	TTSM	3.18800681249	0.00702182686866
k__Bacteria.p__Bacteroidota.c__Cytophagia.o__Cytophagales.f__Fulvivirgaceae.g__Fulvivirga	TTSH	3.45477290083	0.00207071091608
k__Bacteria.p__Bacteroidota.c__Flavobacteriia.o__Flavobacteriales.f__Crocinitomicaceae	TTSH	3.16859862341	4.86970142659E-4
k__Bacteria.p__Bacillota.c__Bacilli.o__Lactobacillales.f__Lactobacillaceae.g__Lactobacillus	NC	3.90182598443	0.0319515219998

k__Bacteria.p__Bacteroidota.c__Flavobacteriia	TTSH	3.06377273436	3.49654879915E-4
k__Bacteria.p__Planctomycetota.c__Planctomycetia.o__Isosphaerales	TTSL	2.73613137636	0.00667973585357
k__Bacteria.p__Actinomycetota.c__Acidimicrobiia.o__Acidimicrobiales	TTSM	3.10722312093	2.36060995306E-4
k__Bacteria.p__Bacteroidota.c__Bacteroidia.o__Bacteroidales	NC	5.12648073338	0.00243188778048
k__Bacteria.p__Planctomycetota.c__Planctomycetia.o__Isosphaerales.f__Isosphaeraceae	TTSL	2.72670543098	0.00667973585357
k__Bacteria.p__Bacteroidota.c__Flavobacteriia.o__Flavobacteriales	TTSH	3.07343931077	3.49654879915E-4
k__Bacteria.p__Cyanobacteriota.c__Cyanophyceae.o__Coleofasciculales.f__Coleofasciculaceae.g__Marmoreocelis	TTSH	3.37660456283	4.86970142659E-4
k__Bacteria.p__Planctomycetota	TTSL	3.24435046392	0.00187615972561
k__Bacteria.p__Planctomycetota.c__Planctomycetia.o__Isosphaerales.f__Isosphaeraceae.g__Singulisphaera	TTSL	3.12084981447	0.00667973585357
k__Bacteria.p__Actinomycetota.c__Actinobacteria.o__Streptosporangiales	TTSL	2.59983371321	1.23092977568E-4

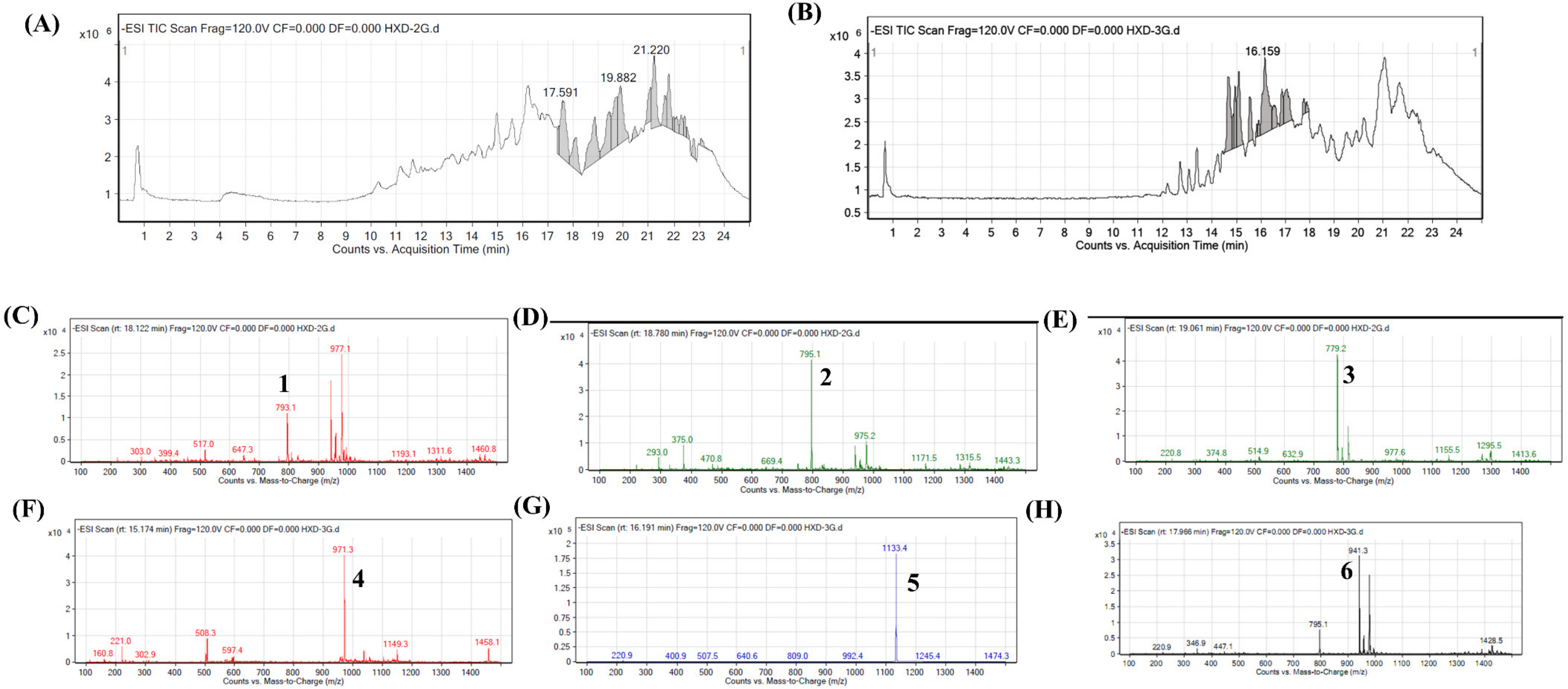

Figure S1. Representative base peak ion chromatograms (BPI) of DTS (A) and TTS (B) extracts acquired in negative ion mode, and the mass spectra of the corresponding peaks **1**–**6** (C–H). The identified compounds and their MS data are listed as follows: **1**. Angulasaponin A (RT: 18.122 min, m/z 977.1); **2**. azukisaponin II (RT: 18.780 min, m/z 795.1); **3**. azukisaponin I (RT: 19.061 min, m/z 779.2); **4**. azukisaponin IV (RT: 15.174 min, m/z 971.3); **5**. azukisaponin VI (RT: 16.191 min, m/z 1133.4); **6**. yunganoside B1 (RT: 13.988 min, m/z 941.3)。

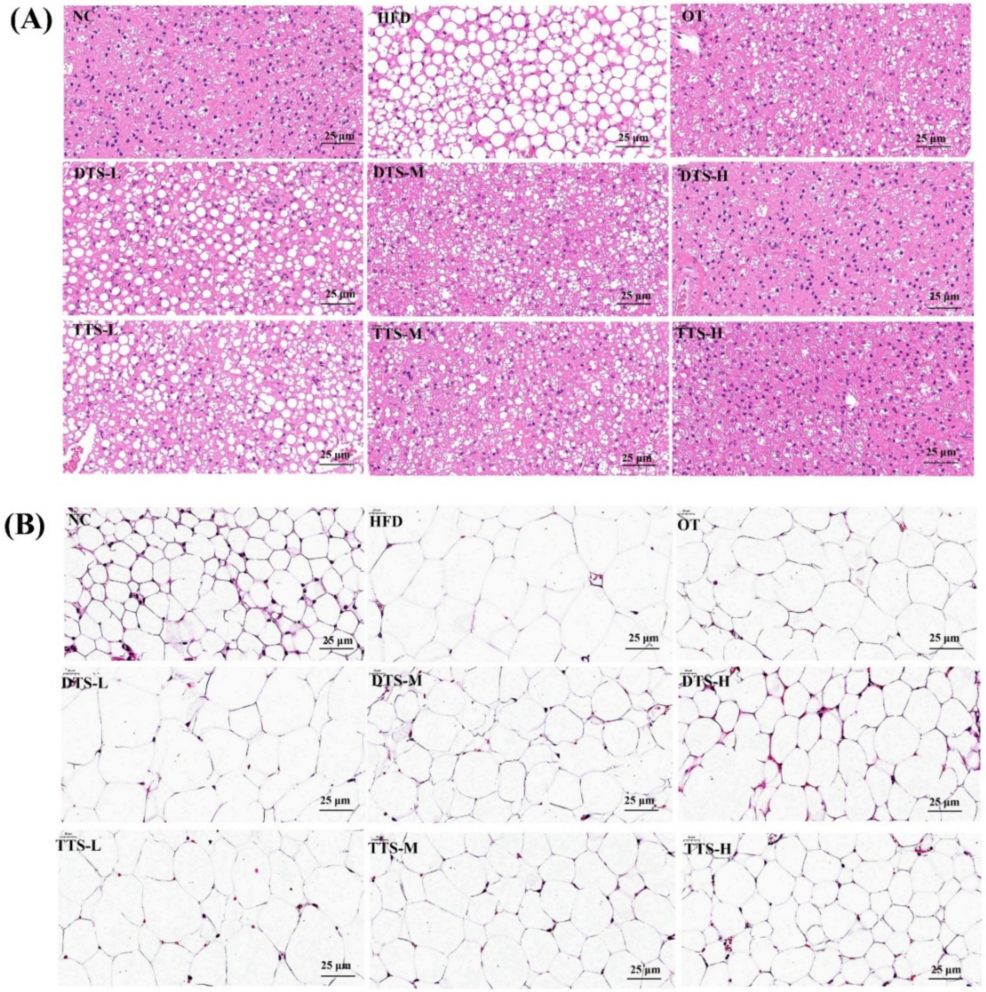

Figure S2. Hematoxylin and eosin (H&E) staining of（A）brown adipose tissue (BAT), and (B) epididymal white adipose tissue (WAT) from mice fed different diets (×40，magnification, 25 μm).

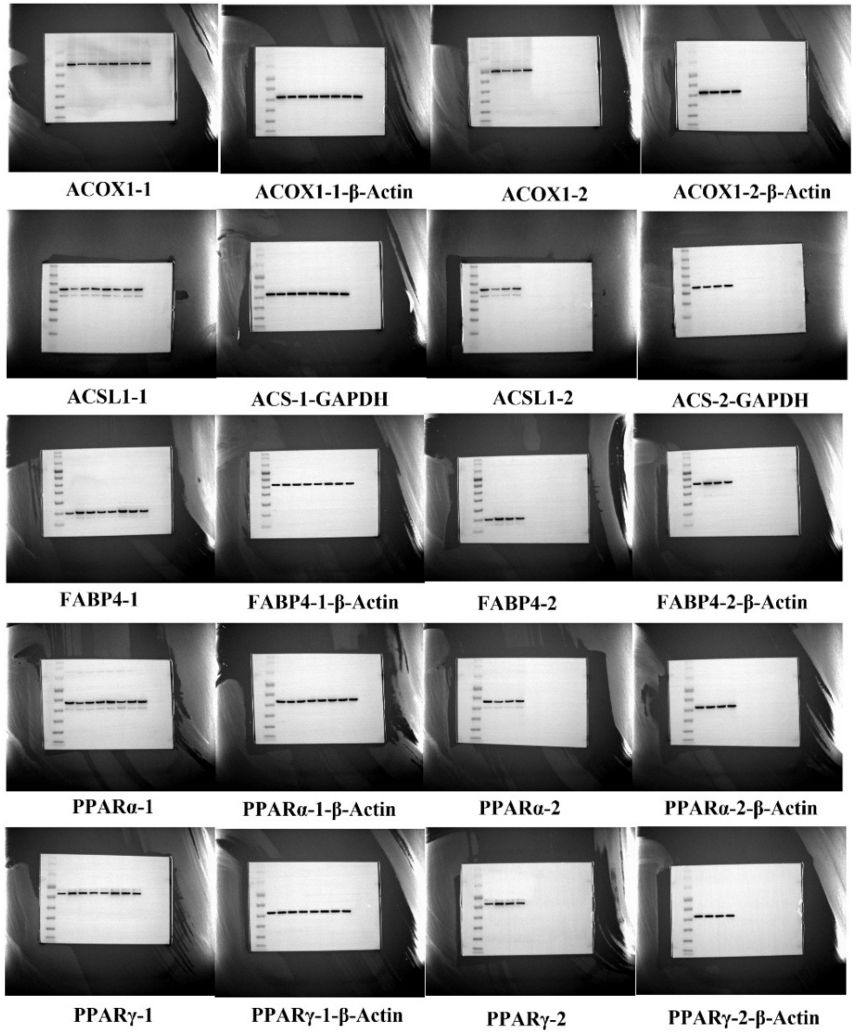

Figure S3 Western blots of ACOX1, ACSL1, FABP4, PPARα, and PPARγ in differentiated 3T3-L1 adipocytes, with β-Actin or GADPH as a loading control.

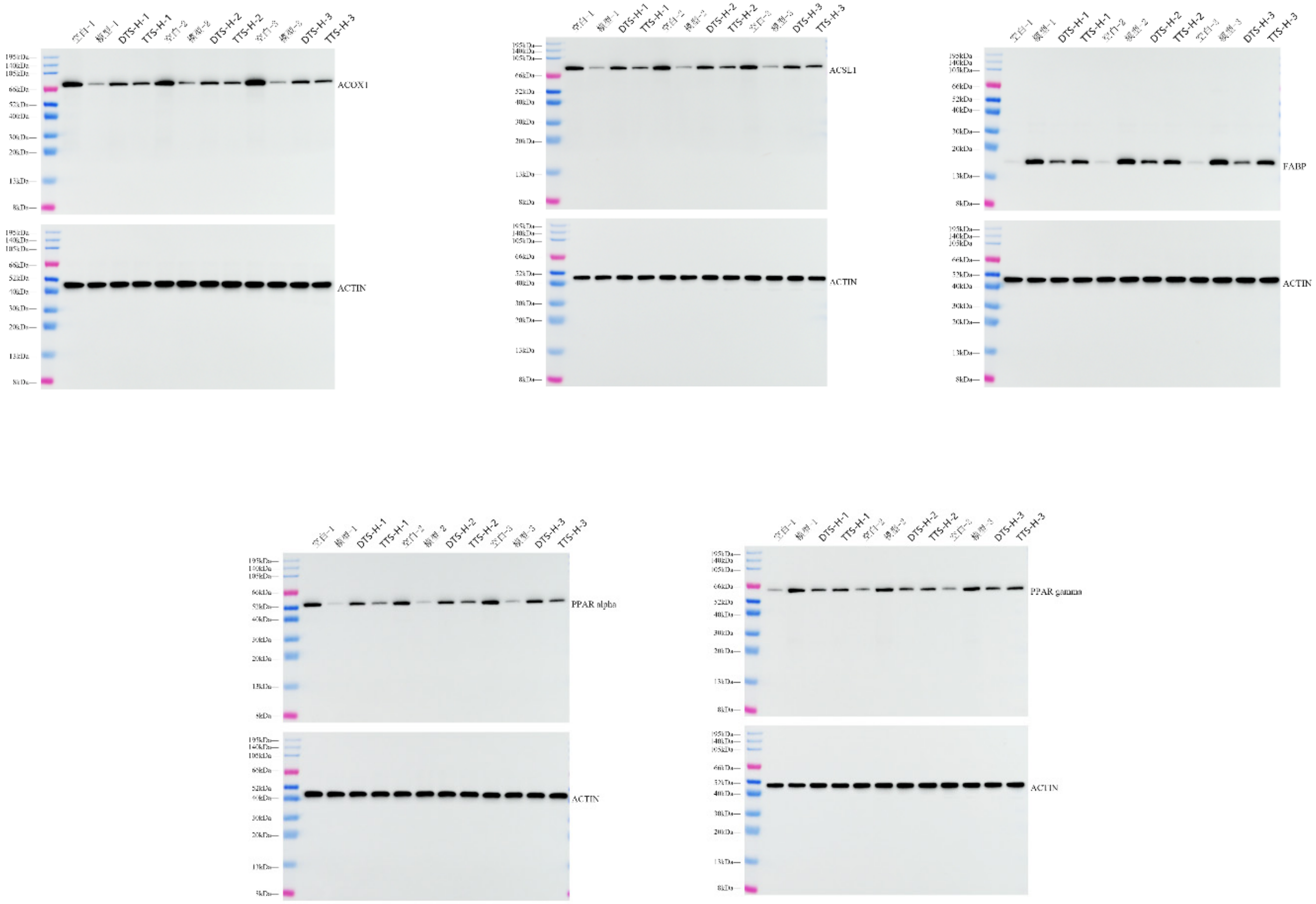

Figure S4 Western blots of AMPK, p-AMPK, FASN, and SREBP1c in HFD-fed mice, with β-Actin as a loading control.
